# A Metalloporphyrin Nanosystem Enables Non‐Invasive Visualization and Specific Treatment for Thrombosis and Ischemic Stroke

**DOI:** 10.1002/advs.202515079

**Published:** 2025-10-05

**Authors:** Ziwei Wang, Liping Zhang, Zihan Wu, Nan Xiao, Wenxin Zheng, Yachao Wang, Gelin Xu, Dongxia Zhu, Martin R. Bryce, Lijie Ren, Ben Zhong Tang

**Affiliations:** ^1^ Key Laboratory of Nanobiosensing and Nanobioanalysis at Universities of Jilin Province Department of Chemistry Northeast Normal University 5268 Renmin Street Changchun Jilin Province 130024 China; ^2^ Department of Neurology Inst Translat Med The First Affiliated Hospital of Shenzhen University Shenzhen Second People's Hospital Shenzhen 518035 China; ^3^ Department of Chemistry Durham University Durham DH1 3LE UK; ^4^ School of Science and Engineering Shenzhen Institute of Aggregate Science and Technology The Chinese University of Hong Kong Shenzhen Guangdong 518172 China

**Keywords:** iridium complex, ischemic stroke, metalloporphyrin, single‐molecule, thrombolysis

## Abstract

Thrombotic ischemic stroke (IS) is a severe neurological disease of the brain, with high global mortality and disability rates. The significant temporal disconnect and lack of coordination between thrombolysis (≥15% hemorrhage risk from short half‐life thrombolytics) and IS treatment results in limited clinical efficacy. Innovative strategies are required to: 1) resolve stereochemical conflicts in single‐molecule multifunctional materials; 2) enable spatiotemporal functions for effective diagnosis and treatment; 3) establish unambiguous structure‐property relationships. Herein, 4IrMn nanoparticles (NPs), with a dual transition metal core exhibit an endogenous peroxynitrite (ONOO^−^) response, which triggers near‐infrared chemiluminescence (NIR‐CL) signals for the precise location of lesion sites. Under laser irradiation, 4IrMn NPs initiate synergistic photothermal therapy (PTT) and photodynamic therapy (PDT) in mouse thrombus models to achieve effective thrombolysis (the relative volume of clots decreased by ≈60%) with no observed hemorrhagic complications or recurrent embolism. After thrombolysis, 4IrMn NPs effectively scavenge excess cytotoxic reactive oxygen/nitrogen species (RONS) and suppresses pro‐inflammatory cytokines such as tumor necrosis factor‐alpha/interleukin‐6 (TNF‐α and IL‐6). Ultimately, 4IrMn NPs enhance neuronal survival in the ischemic penumbra and reduce the extent of cerebral tissue death. This single‐molecule‐based multifunctional nanosystem integrates diagnostic‐therapeutic‐prognosis features, realizing a significant advance in addressing the constraints of current thrombosis and IS treatment.

## Graphical abstract

Thrombotic ischemic stroke is a severe neurological disease of the brain that presents major challenges for effective diagnosis and treatment. Precisely engineered Ir and Mn cores within a single porphyrin framework of 4IrMn nanoparticles enable endogenous ONOO^−^‐ or laser‐activated switching among NIR‐CL imaging, photothermal / photodynamic therapy (PTT/PDT), and neuroprotection, ultimately realizing spatiotemporally controlled thrombolysis and ischemic stroke therapy.

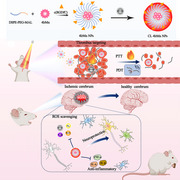

## 1 Introduction

An ischemic stroke (IS) is a medical emergency resulting from cerebral artery occlusion by thrombosis, which leads to inadequate oxygen and blood supply to the brain, causing long‐term disability and death.^[^ ^1^ ^]^ The major goal of IS treatment is to achieve successful reperfusion of blood flow and to salvage the ischemic penumbra in order to minimize the area of tissue death (infarction) and eventually to ameliorate neurological dysfunction.^[^ ^2^ ^]^ Currently, thrombolysis and neuroprotection are two important means for IS intervention.^[^ ^3^ ^]^ Unfortunately, existing thrombolytic drugs usually require high doses for effective treatment due to their short half‐life. However, anterior artery thromboembolism‐induced cerebral infarction results in spatiotemporal dissociation between thrombosis and stroke.^[^ ^4^ ^]^ Excessive thrombolytic therapy may compromise the homeostatic balance of the blood‐brain barrier (BBB) and increase the risk of intracranial hemorrhage.^[^ ^5^ ^]^ These drugs also fail to effectively address cerebral ischemia‐reperfusion injury (CIRI) by blood flow restoration, including excessive cytotoxic reactive oxygen and nitrogen species (RONS) causing oxidative damage that induces secondary neuronal cell death.^[^ ^6^ ^]^ At present, IS monitoring is mostly based on computed tomography and magnetic resonance imaging. There is a lack of effective detection methods for reperfusion biomarkers, making it difficult to provide real‐time and accurate diagnostic information for early‐stage disease.^[^ ^7^ ^]^ Furthermore, diagnostic and therapeutic approaches to thrombosis and IS focus primarily on composite materials that integrate multiple components with different functions into a single platform, which are limited by difficulties in large‐scale preparation with good repeatability, and long‐term toxicity concerns.^[^ ^8^ ^]^ However, developing single‐molecular complexes with well‐defined structure‐activity relationships may increase stereochemical complexity, which presents significant challenges for selectively controlling a single function.^[^ ^9^ ^]^ Therefore, the innovative protocol for a single‐molecule to achieve versatile function with early monitoring of thrombosis/IS and enhanced therapeutic efficacy is fundamentally important, and to date has not been reported.

Nerve cells are more fragile than vascular cells and rapidly lose function under ischaemic conditions.^[^ ^10^ ^]^ Prompt thrombolysis is essential to slow disease progression and to reduce cell death.^[^ ^11^ ^]^ The use of recombinant tissue plasminogen activator (rt‐PA) and urokinase (UK) as an early thrombolytic therapy is limited by their short half‐life, extremely narrow therapeutic window and high risk of hemorrhage.^[^ ^12^ ^]^ In recent years, non‐invasive photothermal therapy (PTT) and photodynamic therapy (PDT) have been effective in inducing copper‐dependent cell death (cuproptosis) for antithrombotic treatment, but there remains room for optimization.^[^ ^13^ ^]^ PTT converts light energy into heat energy through the Landau damping effect, generating localized high temperatures to dissolve fibrin clots.^[^ ^14^ ^]^ However, PTT alone is insufficient to eradicate thrombosis and is accompanied by a high recurrence rate.^[^ ^15^ ^]^ PDT relies on the photoactivation of a photosensitizer (PS) in the presence of oxygen to create ROS. The PS is excited to a singlet state (^1^PS) which then undergoes intersystem crossing (ISC) to a longer‐lived triplet state (^3^PS). The energy of the ^3^PS state is transferred to molecular oxygen (^3^O_2_) to produce singlet oxygen (^1^O_2_) which disrupts the fibrin framework of the thrombus, thus avoiding the risk of secondary embolism (blockage of blood vessels) that may result from PTT therapy.^[^ ^16^ ^]^ Nevertheless, as PDT and PTT utilize the same light excitation energy, it is a challenge to maximize their simultaneous functionality.^[^ ^17^ ^]^ Porphyrin derivatives possess an extensive π‐electron conjugated system and high molar extinction coefficients across the visible to near‐infrared (NIR) spectral regions. They are widely employed as photosensitizers for clinical use, for example Verteporfin.^[^ ^18^ ^]^ However, porphyrin derivatives possess the drawbacks of a larger energy gap (△E_ST_) between the first singlet excited state (S_1_) and triplet state (T_1_) which directly leads to reduced ^1^O_2_ production and low photothermal conversion efficiency.^[^ ^19^ ^]^ The heavy‐atom characteristics of iridium can substantially enhance the spin‐orbit coupling (SOC) effect, facilitatingISC from S_1_ to T_1_, thereby significantly boosting the production of more triplet excited states.^[^ ^20^ ^]^ Developing strategies that balance the requirements between photothermal conversion efficiency and controllable generation of ROS should provide new insights into PTT/PDT dual‐modal materials for thrombus‐specific therapy.

Following thrombolysis and reperfusion (the restoration of blood flow), the abrupt elevation of oxygen partial pressure in ischemic tissues triggers a cascade of pathological reactions: 1) a surge in generation of oxygen free radicals inducing oxidative stress; 2) activation of inflammatory pathways.^[^ ^21^ ^]^ These two processes synergistically expand both neurological deficiency and infarct volume caused by IS. Excessive reactive oxygen species (ROS) cause oxidative damage to DNA, proteins, and lipids, resulting in neuronal cell death constituting the cerebral ischemia‐reperfusion injury (CIRI) which exacerbates the initial tissue damage.^[^ ^22^ ^]^ The core driver is the excessive production of RONS, including peroxynitrite (ONOO^−^), hydroxyl radicals (^•^OH), and superoxide anion (O_2_^−^).^[^ ^23^ ^]^ Transition metal‐based antioxidant enzymes exhibit significant neuroprotective potential by eliminating RONS and mitigating reperfusion injury and the hostile inflammatory microenvironment associated with IS.^[^ ^24^ ^]^ Superoxide dismutase (SOD) and catalase achieve cascade catalytic elimination of ROS through metal active centers.^[^ ^25^ ^]^ Inspired by Mn‐SOD as a prototype, Mn‐coordinated tetrapyrrolic macrocyclic ligand structures have been successfully synthesized as enzyme mimetics.^[^ ^26^ ^]^ These analogs not only retain the catalytic efficiency of natural enzymes but also exhibit enhanced stability.^[^ ^27^ ^]^ The strategic spatial distribution of metal cores within the molecular architecture mitigates potential stereochemical conflicts between metallic centers, posing a challenge in molecular design for achieving synergistic therapeutic efficacy against thrombosis and IS.

Accurate diagnosis and effective therapeutic intervention are equally critical in combating IS.^[^ ^28^ ^]^ Among the harmful RONS, ONOO^−^ has a significantly higher destructive effect on biomolecules due to its dual oxidizing/nitrating ability.^[^ ^29^ ^]^ Therefore, ONOO^−^ is a diagnostic and a prognostic biomarker for IS.^[^ ^30^ ^]^ Despite the well‐established role of ONOO^−^ in IS, effective and selective probes for its real‐time monitoring are lacking.^[^ ^31^ ^]^ Chemiluminescent (CL) probes are being developed for ONOO^−^ detection due to their high selectivity, real‐time visualization capability, and noninvasive nature.^[^ ^32^ ^]^ However, conventional strategies employing Schaap's adamantane‐dioxetane system suffer from short emission wavelengths, which limit deep‐seated pathological monitoring.^[^ ^33^ ^]^ In contrast, NIR light (*λ* 650–1700 nm) offers significantly reduced tissue scattering and absorption coefficients, enabling enhanced penetration depths at the centimeter scale.^[^ ^34^ ^]^ Clearly, there is an urgent need for NIR‐CL imaging to visualize lesion sites. Hence, rational tailored design of multifunctional theranostic agents is crucial for synergistic therapy of thrombosis and IS.

**Scheme 1:**
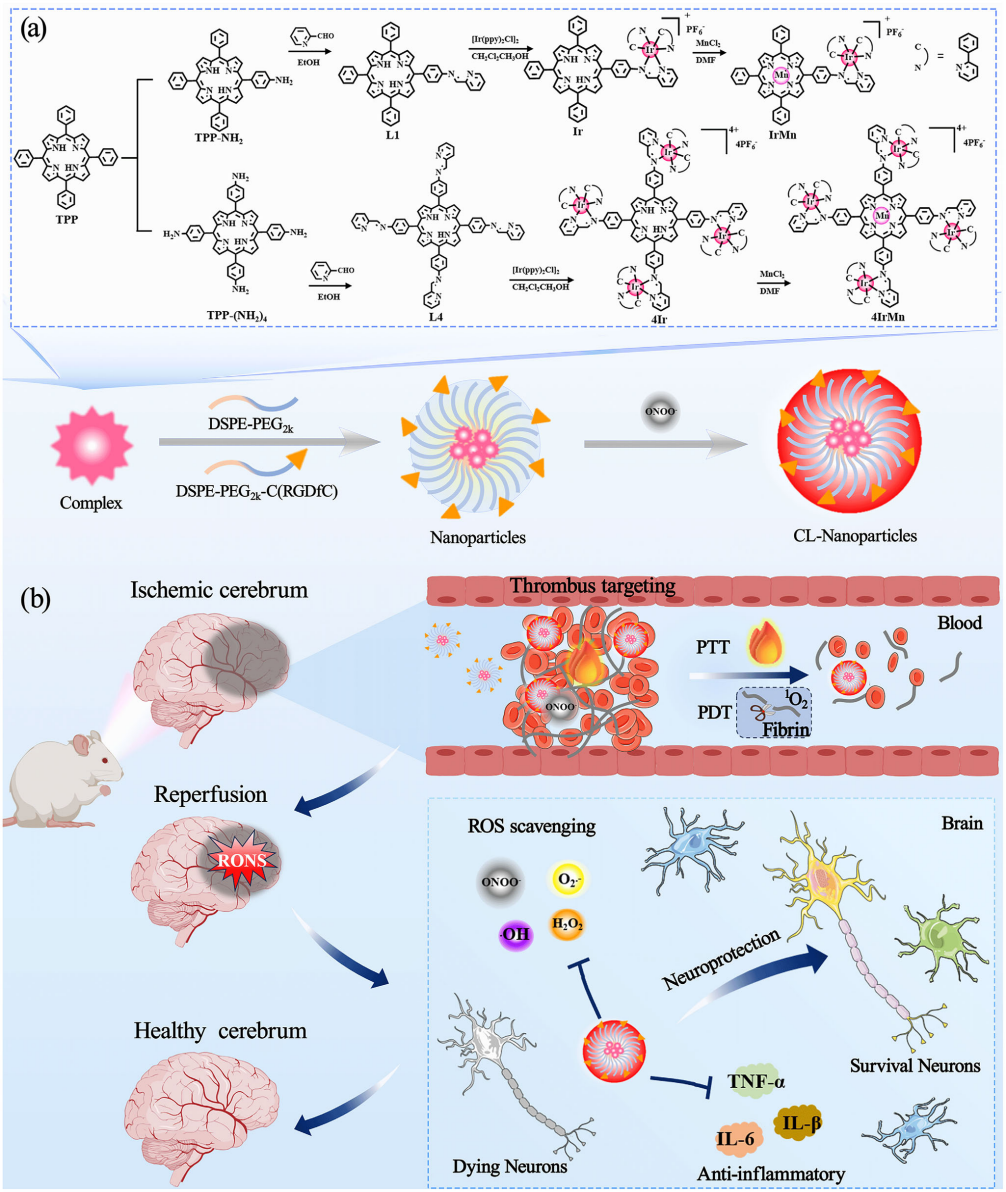
a) Schematic diagram of the structures of the complexes and the preparation of the nanoparticles. b) Schematic diagram of thrombolytic and IS treatment.

In this work, we report a single‐molecule‐based nanosystem (named 4IrMn) that resolves the conflicts among visual lesion localization, highly efficient thrombolysis, and neuroprotection, with on‐demand switching among these modalities achieved via endogenous ONOO^−^ response or by laser activation. 4IrMn is based on a Mn‐coordinated tetraphenylporphyrin (TPP) derivative with four peripheral cyclometalated iridium complexes. The molecule integrates photo‐switchable modalities eliciting thrombolytic effects under laser irradiation while autonomously switching to a neuroprotection mode without laser. This nanosystem resolves the stereochemical conflicts arising from selectively controlling the functionalities of distinct metal cores within a single molecule, while achieving spatiotemporal switching via laser irradiation to realize thrombosis and IS. First, the 4IrMn nanoparticles (NPs) enable precise lesion localization by triggering CL through a specific response to endogenous peroxynitrite (ONOO^−^). Second, due to the conjugation of TPP and cyclometalated Ir(III) complexes, 4IrMn NPs exhibit combined PDT/PTT performance and achieve a reperfusion rate of ≈69.3% on receiving a 635 nm laser stimulus, with no hemorrhage or re‐embolization risks. Third, 4IrMn NPs significantly boost the molecule's enzymatic‐like radical scavenging capacity due to the porphyrin's Mn^2^⁺ coordination. Post‐thrombolysis, the 4IrMn NPs clear excess RONS (O_2_^−^, ^•^OH) generated during reperfusion, reducing pro‐inflammatory cytokine (TNF‐α and IL‐6) levels, while increasing ischemic penumbra neuronal survival by 3.8‐fold and ultimately restoring cerebral infarct volume to 86.1% of the baseline (**Scheme** **1**). This work highlights the potential of a single‐molecule‐based nanosystem as a versatile solution for spatiotemporal treatment of both thrombosis and IS, paving the way for more advanced biomedical applications.

## 2 Results and Discussion

The synthetic pathways for the complexes investigated in this study are illustrated in Scheme S1 (Supporting Information). Initially, Ir and 4Ir complexes were synthesized following established protocols,^[^ ^35^ ^]^ after which they were reacted with an excess of MnCl_2_ in DMF solvent to obtain the two bimetallic complexes, IrMn and 4IrMn. Mass spectra of IrMn and 4IrMn are shown in Figures S1 and S2 (Supporting Information). The successful synthesis of IrMn and 4IrMn is also evidenced by the disappearance of the N─H bending vibrational peak in the porphyrin ring at 966 cm^−1^ in the FT‐IR spectra as shown in Figure S3 (Supporting Information), and the appearance of the N‐Mn metal‐sensitive peak at 1010 cm^−1^. Water‐soluble nanoparticles were then synthesized by the classical nanoprecipitation method.^[^ ^36^ ^]^ The hydrophobic part of the amphiphilic polymer DSPE‐PEG‐MAL was wrapped around 4IrMn with the hydrophilic end facing outwards to make it uniformly dispersed in the aqueous phase. Subsequently, the peptide c(RGDfC) was attached through a click reaction to obtain the 4IrMn NPs; the disappearance of the maleimide group signal at δ 7.0 ppm indicated the successful conjugation of c(RGDfC) to DSPE‐PEG‐MAL. Other nanoparticles were produced by the same method (as described in Figure S4, Supporting Information).

**Figure 1:**
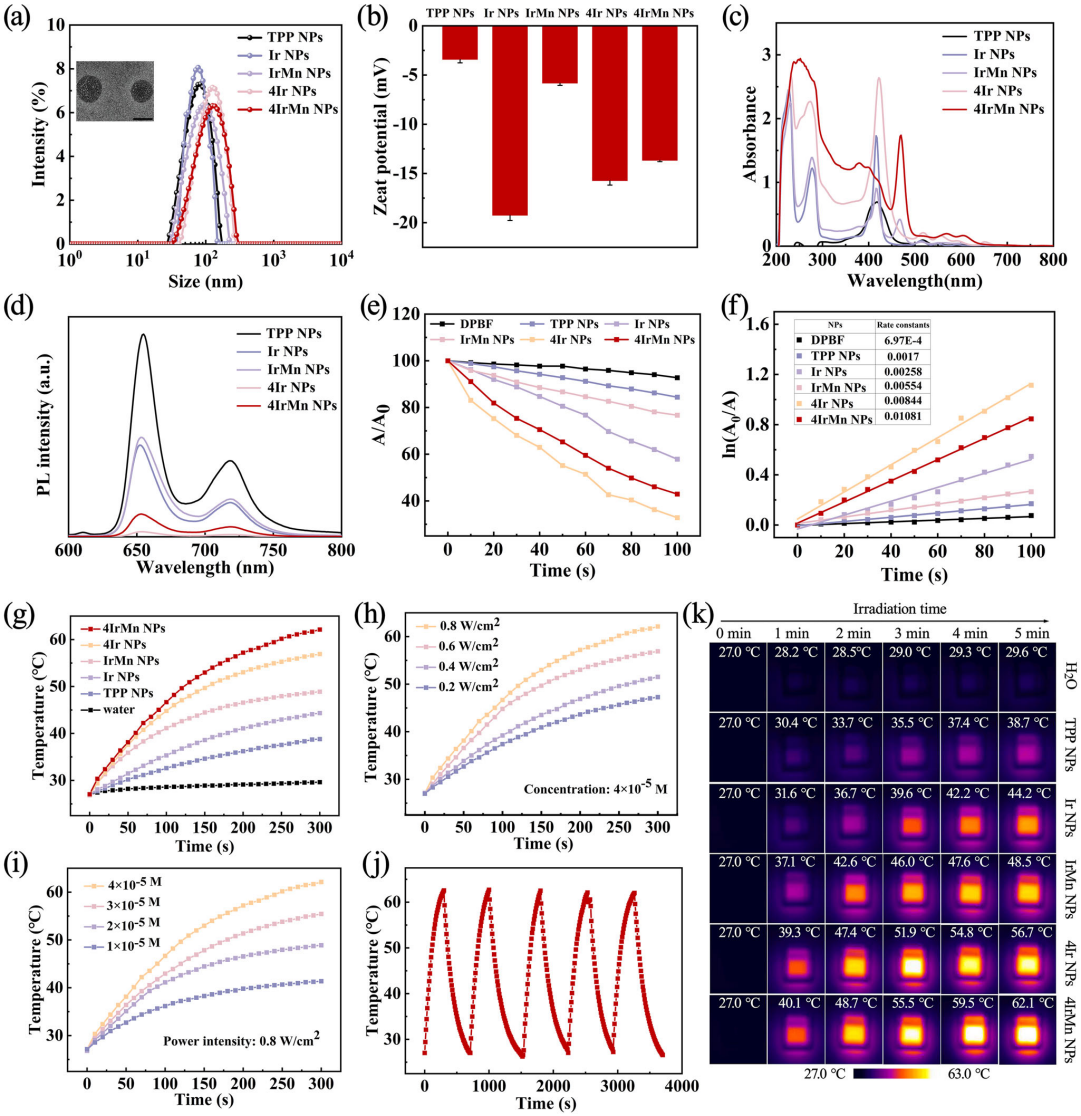
Structural and physical properties characterizations of the NPs. a) The mean hydrodynamic size and b) Zeta potential of TPP NPs, Ir NPs, IrMn NPs, 4Ir NPs, and 4IrMn NPs (the inset is the TEM image of 4IrMn NPs in Figure 1a, Scale bar = 100 nm). c) UV–vis absorption d) Fluorescence spectra of TPP NPs, Ir NPs, IrMn NPs, 4Ir NPs, and 4IrMn NPs in water. e) Comparison of decay rates of TPP NPs, Ir NPs, IrMn NPs, 4Ir NPs, and 4IrMn NPs under irradiation (635 nm, 0.8 W cm^−2^), A_0_ = DPBF absorption under no irradiation conditions. A = Real‐time absorption of DPBF at different irradiation times. f) ^1^O_2_ generation kinetics over time. g) Photothermal heating curves of TPP NPs, Ir NPs, IrMn NPs, 4Ir NPs, and 4IrMn NPs under irradiation of 635 nm laser. h) Laser power density and i) concentration dependence of the photothermal effects of 4IrMn NPs under 635 nm laser irradiation. j) Temperature change of the 4IrMn NPs solution during five laser irradiation cycles. k) Infrared images of NPs under laser irradiation.

Dynamic light scattering (DLS) analysis revealed that the complexes formed nanoscale aggregates with hydrodynamic diameters of ≈79 nm (TPP NPs), 80 nm (Ir NPs), 84 nm (IrMn NPs), 125 nm (4Ir NPs), and 131 nm (4IrMn NPs) (**Figure** **1**a). Transmission electron microscopy (TEM) further corroborated these findings, confirming nanometer‐sized aggregates (Figure 1a; Figure S5, Supporting Information). The DLS and polydispersity index of all the NPs remained almost unchanged after 14 days of storage as shown in Figure S6 (Supporting Information), showing good long‐term stability. Zeta potential measurements indicated that all the NPs in water exhibited similarly strong negative charges (Figure 1b), which are conducive to stable circulation and transportation within the body. The absorption and fluorescence emission spectra of the NPs (10 µm in deionized water) are shown in Figures 1c,d. The pronounced absorption enhancement of 4IrMn NPs at 635 nm underpins the selection of the laser light source.

The photophysical properties of the NPs were explored. Their ^1^O_2_ generation ability was monitored by using 1,3‐diphenylisobenzofuran (DPBF) as an indicator. Upon irradiation of DPBF solutions in the presence of TPP NPs, Ir NPs, IrMn NPs, 4Ir NPs, and 4IrMn NPs (10^−5^ M in water), the absorption peak intensity of DPBF at 415 nm gradually decreased (Figures S7–S10, Supporting Information), confirming the efficient ^1^O_2_ generation by these NPs. As shown in Figures 1e,f, ^1^O_2_ generation follows first‐order kinetics. A steeper slope represents a quicker decay rate of DPBF and higher ability to generate ^1^O_2_. A trend is that the introduction of Mn results in slightly lower ^1^O_2_ yields for IrMn NPs and 4IrMn NPs than for Ir NPs and 4Ir NPs. This could be attributed to the quenching effect of the paramagnetic Mn(II) center on the excited state. The calculated ^1^O_2_ generation efficiency of Rose Bengal under the same conditions was 63%, while 4IrMn NPs exhibited a slightly higher ^1^O_2_ generation efficiency of 68.9%, which will be favorable for subsequent PDT thrombolysis. Then the photothermal features of the NPs under 635 nm laser irradiation were explored. At a power density of 0.8 W cm^−2^, the temperature of the dispersion of 4IrMn NPs could reach more than 60 °C after 5 min, which was sufficient to effectively dissolve the thrombus block (Figure 1g). Figures 1h,i presents the heating curves of 635 nm laser irradiation under different power densities (0.2, 0.4, 0.6and 0.8 W cm^−2^) and concentrations (10^−5^, 2 × 10^−5^, 3 × 10^−5^, and 4 × 10^−5^ M in water) for 4IrMn NPs, demonstrating that the temperature of the solutions of the NPs clearly increases with enhancing the concentrations and laser power density. The test results showed that when the laser power was 0.8 W cm^−2^ and the concentration was 4 × 10^−5^ M, the photothermal effect of 4IrMn NPs could reach its optimum. Therefore, we determined the conditions for the photothermal test. 4IrMn NPs also have good photothermal stability even after five cycles of heating and cooling (Figure 1j). The photothermal conversion efficiency of 4IrMn NPs is 57.5% (Figure S11, Supporting Information). Figure 1k shows IR thermal images of the NPs (40 µm) under laser irradiation (635 nm, 0.8 W cm^−2^) at different exposure times. These results establish that 4IrMn NPs possess excellent PDT and PTT efficiencies, which is conducive to achieving high‐efficiency thrombolysis.^[^ ^37^ ^]^

**Figure 2:**
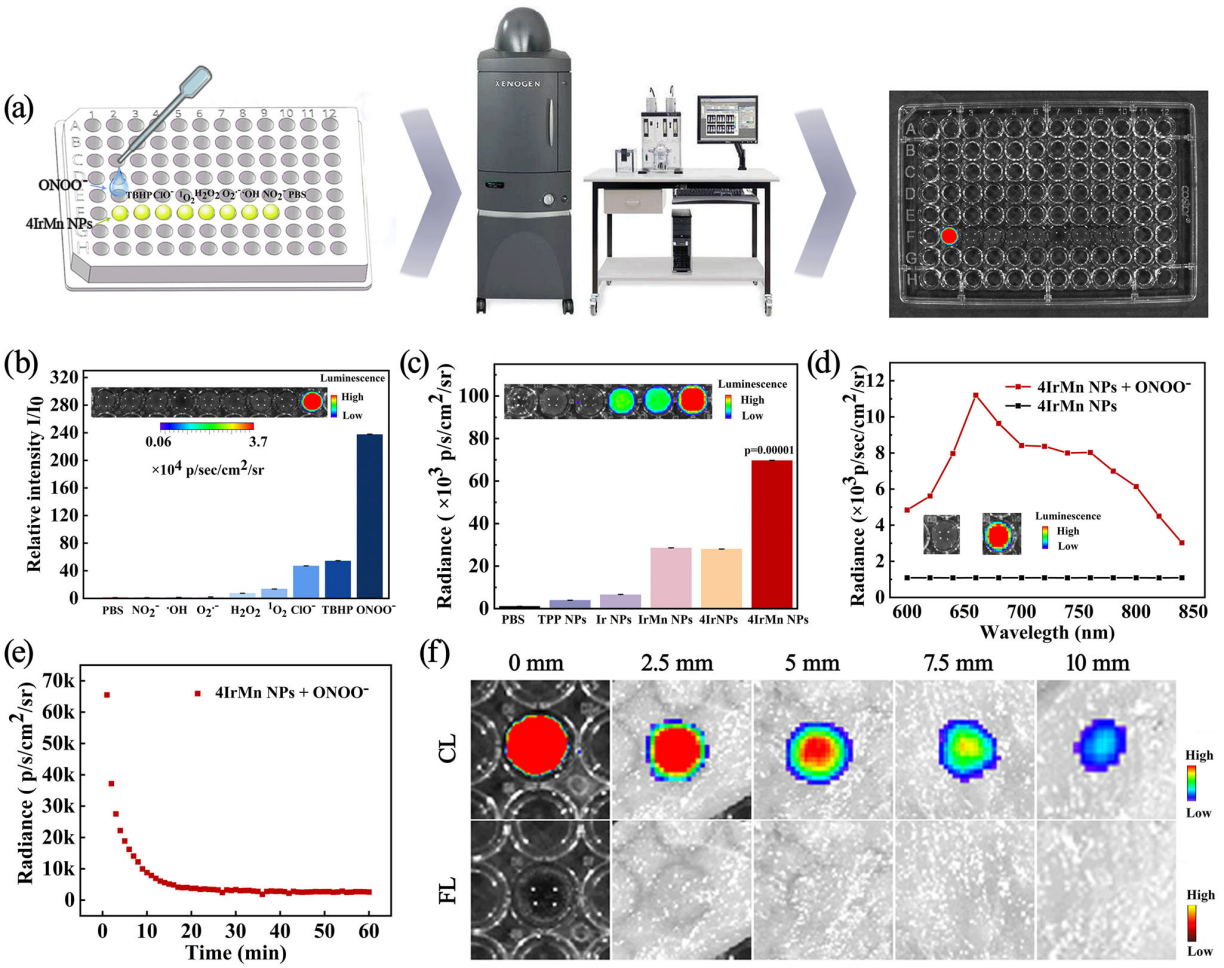
Characterization of nanoparticle CL properties. a) The process of reagent measurement of CL. b) Selectivity of the 4IrMn NPs toward different RONS (200 µm) treatments. c) CL intensity plot of TPP NPs, Ir NPs, IrMn NPs, 4Ir NPs, and 4IrMn NPs in the presence of ONOO^−^ (200 µm). d) CL spectra of 4IrMn NPs in the presence or absence of ONOO^−^ (200 µm) in PBS solution (pH 7.4). e) Decay of persistent luminescence signal of 4IrMn NPs (200 µg mL^−1^) over time at room temperature of ONOO^−^ (200 µm). f) CL signals of 4IrMn NPs (in PBS at pH 7.4) added to ONOO^−^ covered with different thicknesses of chicken breast tissue. Inset: the CL images acquired by an IVIS imaging system under bioluminescence mode with an open filter. Data are presented as mean ± SD (*n* = 3 biological replicates). The data were analyzed by unpaired 2‐tailed Student's t‐test using the GraphPad Prism 7 (c). *p* < 0.05 was considered statistically significant. *p < 0.05, **p < 0.01, and ***p < 0.001 versus control group.

Chemiluminescence (CL) does not require an external light source, instead CL is generated by in situ chemical reactions, thereby leading to minimized tissue autofluorescence and enhanced imaging sensitivity. The process of reagent measurement is shown in **Figure** **2**a. CL has emerged as a groundbreaking method for biological imaging. Zhu et al. have reported that Ir complexes with Schiff base structures can react with ROS through a unique intramolecular imine to amide conversion; the resulting CL reaction was rapid, with a half‐life of only 0.86 s, significantly faster than previous examples. However, the longest half‐life of CL reactions is 34.7 s, which is too short for long‐term tracking of the lesion site.^[^ ^38^ ^]^ We have now integrated Ir complexes with porphyrins,^[^ ^39^ ^]^ both of which are capable of generating CL, to synthesize 4IrMn NPs exhibiting a significantly prolonged CL half‐life.

First, a series of RONS, namely, NO_2_^−^, *t*‐Bu‐hydroperoxide (TBHP), OH^−^, O_2_^•^^−^, ^1^O_2_, H_2_O_2_, ClO^−^and ONOO^−^, were used to investigate the selectivity of the CL of 4IrMn NPs (Figure 2b; Figure S12, Supporting Information). A pronounced CL signal was detected in the presence of ONOO^−^, confirming the highly selective recognition of ONOO^−^. The signal intensity was enhanced ≈237‐fold relative to phosphate‐buffered saline (PBS). ONOO^−^ also excited CL in the other NPs, but the 4IrMn NPs exhibited the most significant intensity, measuring 18 times greater than that of the TPP NPs (Figure 2c; Figure S13, Supporting Information). The CL spectrum of 4IrMn NPs shows a prominent and broad emission peak in the range of 600–850 nm, demonstrating excellent NIR‐CL (Figure 2d). The persistent CL signal indicated >10 min photon release (Figure 2e) sufficient for in vivo imaging and drug screening purposes. The persistent CL signal of 4IrMn NPs showed a linear correlation with ONOO^−^ concentration (Figure S14, Supporting Information). The pH values ranging from 5.0 to 8.5 have little effect on the chemiluminescence intensity of 4IrMn NPs, which proves that 4IrMn NPs are suitable for chemiluminescence imaging in different acidic and alkaline environments (Figure S15, Supporting Information). The chemiluminescence of 4IrMn NPs is similar to their fluorescence spectra (Figure S16, Supporting Information) as observed in previous literature.^[^ ^38^ ^]^ Deep tissue penetration is crucial for the effective use of NPs in brain imaging. When the reaction mixture was covered with chicken breast tissue the intensity of NIR‐CL signal diminished as the thickness of the chicken tissue increased. However, the signal remained clearly detectable even in tissues up to 10 mm thick (Figure 2f).

**Figure 3:**
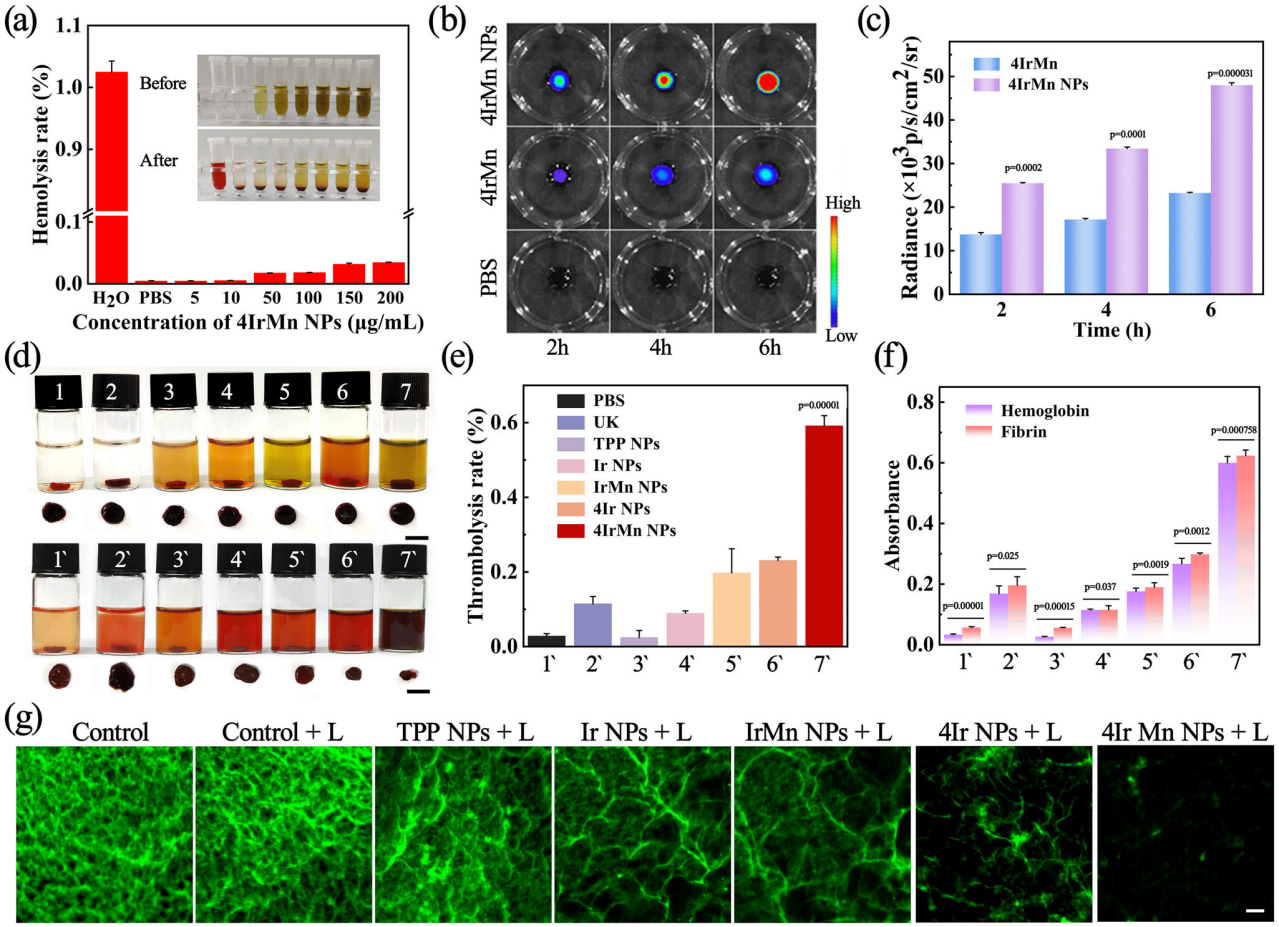
a) The hemolysis rate of red blood cells treated with water, PBS, and different concentrations of 4IrMn NPs. Inset shows the color change of the solution before and after the red blood cells were added (from left to right: water, PBS, 5, 10, 50, 100, 150and 200 µg mL^‒1^ of 4IrMn NPs). Data are presented as mean ± SD (*n* = 3 biological replicates). b) The CL images of artificial blood clots after incubation with PBS, 4IrMn, or 4IrMn NPs for different times. Scale bar = 5 mm. c) Quantitative analysis of the relative CL intensity of the artificial blood clots in (Figure 3b). d) Photographs of the blood clot solution after different treatments. (The pictures above are 1: PBS, 2:UK, 3: TPP NPs, 4: Ir NPs, 5: IrMn NPs, 6: 4Ir NPsand 7: 4IrMn NPs the pictures below correspond to the light groups, respectively). The residual blood clots were taken out and are shown below. Scale bars = 5 mm. e) The quantified clot–dissolution efficiency of different treatment groups (corresponding to Figure 3d) in vitro. f) The absorbance at 450 and 540 nm in different treatment groups (corresponding to Figure 3d) after different treatments. g) CLSM images of fibrin clot after incubating: “Control” without irradiation or NPs with irradiation (L), and TPP NPs, Ir NPs, IrMn NPs, 4Ir NPs, 4IrMn NPs with irradiation. Scale bar = 100 µm. Data are presented as mean ± SD (*n* = 3 biological replicates). p < 0.05 was considered statistically significant. The data were analyzed by unpaired 2‐tailed Student's t‐test using the GraphPad Prism 7 (c, eand f). *p < 0.05, **p < 0.01, and ***p < 0.001 versus the control group.

As the NPs have excellent photophysical properties, a preliminary evaluation of their thrombolytic properties was carried out. Initially, the hemolytic effect of the 4IrMn NPs was examined, and the results illustrate a hemolysis rate of < 5% when co‐cultured with various concentrations of 4IrMn NPs, demonstrating excellent compatibility with blood (**Figure** **3**a). The synthetic blood clots were created by combining fresh blood from mice with thrombin. After the incubation of artificial clots with non‐targeted 4IrMn or targeted 4IrMn NPs for 2, 4and 6 h, the clots were imaged under an in vivo imaging system (IVIS). As anticipated, the blood clots treated with 4IrMn NPs displayed significantly enhanced CL compared to the 4IrMn and PBS groups at each time point (Figures 3b,c), indicating an improved targeting capability facilitated by the c(RGDfC) peptide in the NPs. To evaluate the in vitro thrombolytic effect, the NPs with fresh blood clots were incubated and irradiated with a 635 nm laser. The supernatant of the solution containing the NPs produced obvious turbidity after irradiation, and the volume of the blood clot was significantly reduced (Figure 3d). The results show that the PTT/PDT generated by local light‐activated NPs can rapidly increase the temperature and increase the production of ROS, which promotes thrombolysis. The thrombolysis efficiency was quantified by weighing the mass of the clot before and after the treatment.

Compared with groups PBS, PBS + L, UK, TPP NPs + L, Ir NPs + L, IrMn NPs + Land 4Ir NPs + L (L = light) the dissolution efficiency of 4IrMn NPs + L treatment was the highest, ≈59.1% (Figure 3e). The amount of hemoglobin and fibrin released into the supernatant during clot lysis showed a similar trend to lysis efficiency in Figure 3f. ROS disrupt the fibrin skeleton, as assessed by fluorescein isothiocyanate (FITC)‐labeled fibrin clots assays. In Figure 3g, the quantity of disintegrated fibrin matrix and particulate debris was identified following the incubation of fibrin with NPs under laser irradiation. In the 4IrMn NPs + L treatment group, the degradation of fibrin fragments was the highest. These results demonstrate that 4IrMn NPs have the best thrombolytic capacity in the series, heralding their excellent potential for in vivo therapy.

**Figure 4:**
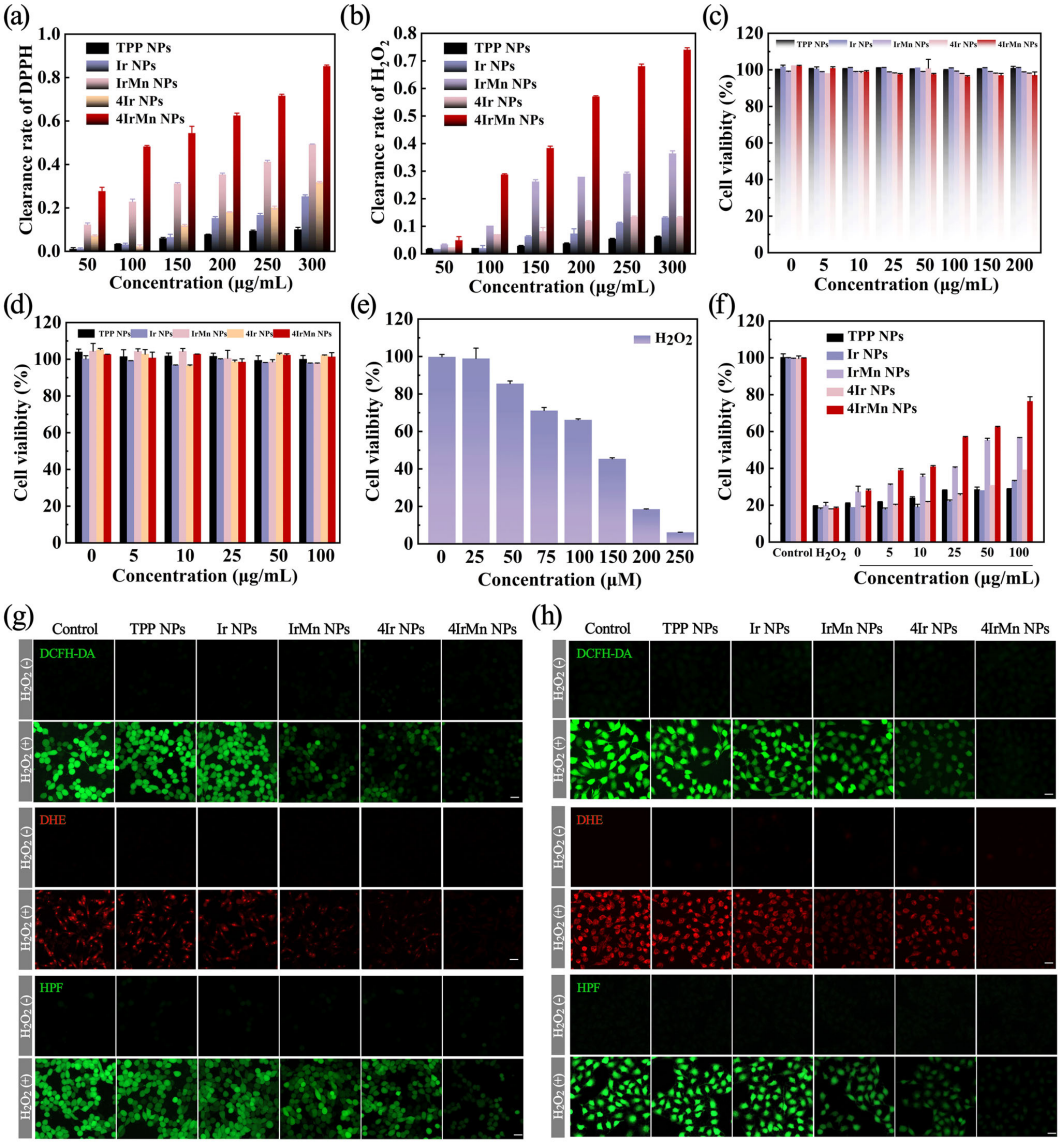
a) DPPH and b) H_2_O_2_ scavenging activity of TPP NPs, Ir NPs, IrMn NPs, 4Ir NPs, and 4IrMn NPs. c) Cell viability was measured after HUVEC and d) HT22 cells were treated with different concentrations of NPs for 24 h. e) Cell viability was measured after HT22 cells were treated with different concentrations of H_2_O_2_. f) Cell viability was measured after HT22 cells were treated with H_2_O_2_ (200 µm) for 24 h and then incubated with different concentrations of NPs for another 24 h. g) 4IrMn NPs scavenged RONS (including O_2_^•−^ and ^•^OH) activity induced by H_2_O_2_ treated HT22 cells in vitro, Scale bar = 50 µm. h) 4IrMn NPs scavenged RONS (including O_2_^•−^ and ^•^OH) activity induced by H_2_O_2_ treated HUVEC cells in vitro, Scale bar = 50 µm.

To assess the antioxidant potential of the NPs, ROS‐scavenging assays were conducted in vitro using DPPH (1,1‐diphenyl‐2‐picryhydrazyl) and H_2_O_2_. By comparing the absorbances of the NP‐containing mixtures with different concentrations and a control solution, the scavenging activities of the NPs were calculated. **Figures** **4**a,b show that an increase in the concentration of 4IrMn NPs from 50 to 100, 150, 200, 250and 300 µg mL^−1^ resulted in an enhanced scavenging efficacy. In a dose‐dependent manner, the 4IrMn NPs significantly inhibited the DPPH and H_2_O_2_ and scavenged up to 80% and 74% radicals at the concentration of 300 µg mL^−1^. This result suggests that the NPs have a ROS response/scavenging property that is predominantly derived from their coordinated transition metals.

To test the in vitro cytotoxicity of the NPs, the viability of human umbilical vein endothelial cells (HUVECs) was studied after treatment with the NPs for 24 h (Figure 4c). The survival rate of the cells exceeded 95% at a concentration of 200 µg mL^−1^ of NPs. In contrast, HT22 cells (a mouse hippocampal cell line) maintained a survival rate of not less than 95% at a concentration of 100 µg mL^−1^ of NPs (Figure 4d), which may be because mouse neural cells are more fragile than vascular cells. These findings indicate that elevated levels of NPs did not induce significant cytotoxic effects on the cells and they have good biocompatibility. As shown in Figure S17 (Supporting Information), free Ir/Mn ions have significant cytotoxicity and cannot be used for in vivo testing. Therefore, free Ir/Mn ions were not appropriate as a control group. Subsequently, to evaluate the neuroprotective effects of the NPs in vitro, HT22 cells were first incubated with different concentrations of H_2_O_2_ for 24 h. The HT22 cells exhibited dose‐dependent cytotoxicity in response to H_2_O_2_, with ≈20% cell viability observed at a concentration of 200 µm (Figure 4e). Next, the cell viability of H_2_O_2_ (200 µm)‐treated cells significantly increased to 76.3% after pretreatment with 4IrMn NPs compared with other NPs groups (Figure 4f). 4IrMn NPs exhibit excellent ROS clearance performance, suggesting itspotential use of ROS scavenging in brain for ischemic stroke treatment (Table S1, Supporting Information). In the context of RONS, ^•^OH and O_2_^•−^ are crucial pathogenic agents in CIRI. We used 2′,7′‐dichlorodihydrofluorescein diacetate (DCFH‐DA) to evaluate total intracellular ROS, and dihydroethidium (DHE) and hydroxyphenyl fluorescein (HPF) were used to assess intracellular O_2_^•−^ and ^•^OH concentrations. As anticipated, the 4IrMn NPs significantly reduced the intracellular concentrations of ROS in cells in a dose‐dependent manner when compared to the control group (Figures 4g,h). The quantitative analysis of the fluorescence intensity is shown in Figures S18–S20 (Supporting Information). The combination of these results revealed that 4IrMn NPs could directly protect the neurons from damage by scavenging ROS and reducing the level of oxidative stress. Meanwhile, the progression of cerebral I/R injury is closely linked to a pronounced inflammatory response. To assess the impact of 4IrMn NPs on key inflammatory mediators in vitro, we conducted experiments following the administration of 1 µg mL^−1^ of lipopolysaccharides (LPS) to RAW 264.7 cells for a duration of 24 h. The findings revealed that LPS triggered a significant inflammatory response. Furthermore, while the 4IrMn NPs demonstrated minimal influence on the levels of TNF‐α and IL‐6 on their own, preincubation with 4IrMn NPs prior to LPS exposure led to a marked reduction in the levels of these inflammatory factors (Figure S21, Supporting Information). This suggests that 4IrMn NPs can effectively mitigate the inflammatory responses in LPS stimulated cells in vitro. These results validate that 4IrMn NPs can reduce pro‐inflammatory factors and modulate the inflammatory microenvironment, which would achieve a synergistic neuroprotective effect with its antioxidant properties for the treatment of IS.

**Figure 5:**
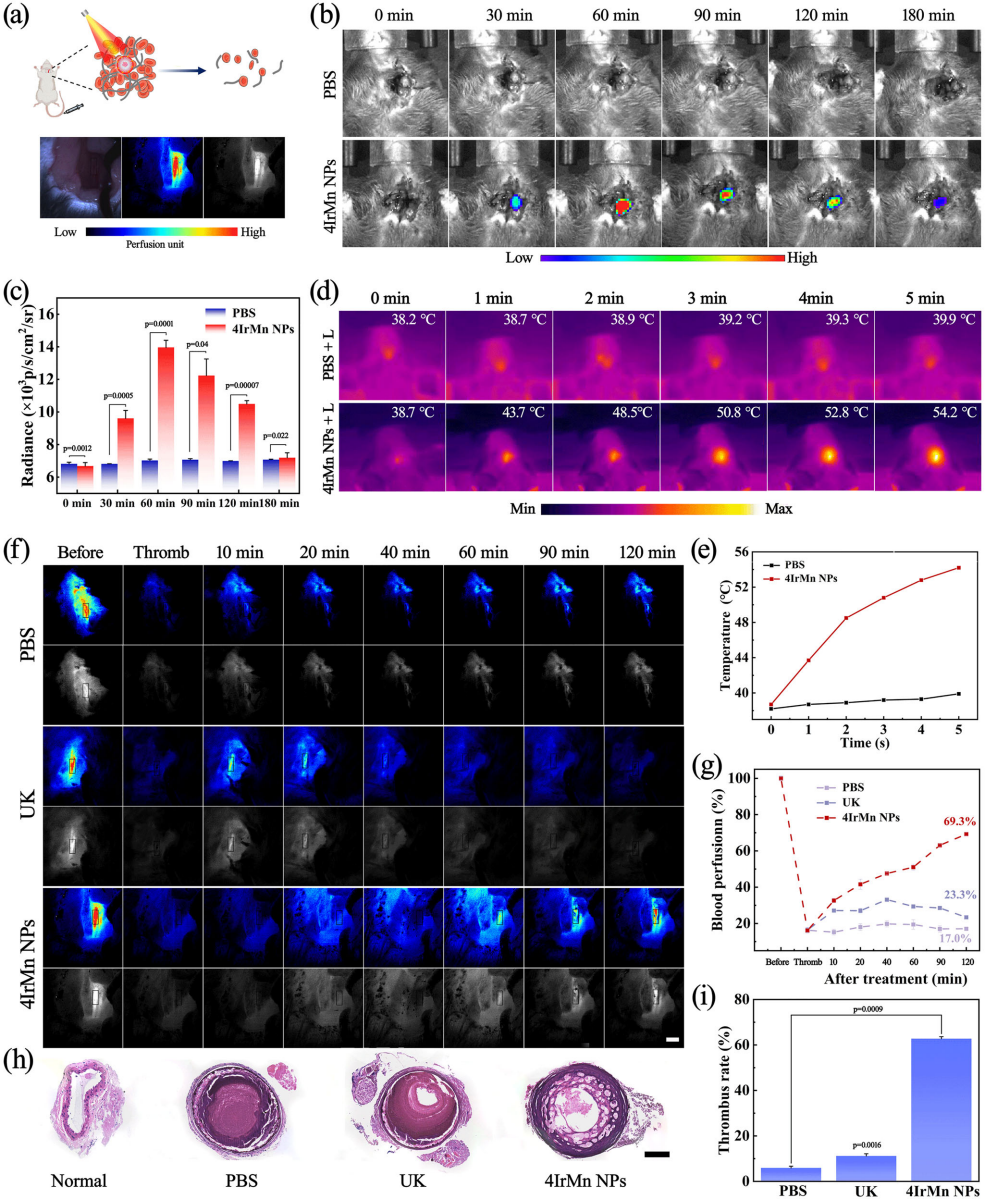
a) Schematic diagram of in vivo thrombolysis, pictured below are LSBFMS images. b) Representative in vivo CL images of the thrombotic artery at different timepoints post‐injection of PBS and 4IrMn NPs. Scale bar = 5 mm. c) Quantification of the relative CL intensity of mouse neck thrombosis in (b). d) Thermal images of the thrombosis site after 635 nm laser (0.8 W cm^−2^) irradiation for different times. Scale bar = 3 mm. e) Thrombus light‐heat curves corresponding to (d). f) During LSBFMS imaging, the right carotid thrombotic artery of each mouse (indicated with black lines) was focused and its blood perfusion was quantified. Scale bars = 5 mm. g) Representative LSBFMS images and the corresponding relative blood perfusion of the mouse carotid artery after FeCl_3_ induction and different therapeutic treatments, including PBS, free UK, 4IrMn NPs. h) Images of H&E staining of the blood vessels and enlarged pictures of thrombus. Scale bar = 200 µm. i) Quantitative statistics of thrombosis area for different groups. Data are presented as mean ± SD (*n* = 3 biological replicates). *p* < 0.05 was considered statistically significant. The data were analyzed by unpaired 2‐tailed Student's t‐test using the GraphPad Prism 7 (c and i). *p < 0.05, **p < 0.01, and ***p < 0.001 versus control group.

Subsequently, the potential of 4IrMn NPs for thrombus theranostics was evaluated in live mice (**Figure** **5**a). A carotid thrombosis model was induced by ferric chloride (FeCl_3_) which relies on the oxidative injury inflicted on vascular endothelial cells by FeCl_3_ and has been extensively utilized for investigating thrombosis and evaluating the efficacy of anti‐thrombotic medications.^[^ ^40^ ^]^ The images obtained from a laser speckle blood flow monitoring system (LSBFMS) clearly indicated the onset of thrombus formation, evidenced by the cessation of blood perfusion. Following the induction of thrombosis, 4IrMn NPs were administered intravenously into the mice through the tail vein. Then the CL signals from the carotid artery were captured at various time intervals using an IVIS system. The signal intensity progressively increased, peaking at 60 min, as the circulating 4IrMn NPs accumulated at the thrombus site (Figures 5b,c). In the groups that underwent laser treatment, the carotid arteries of the mice were exposed to a 635 nm laser (0.8 W cm^‒2^) for 5 min, beginning 60 min after the administration of the 4IrMn NPs. First, a thermal infrared camera monitored temperature changes at the thrombosis sites during the NIR light exposure (Figures 5d,e). The 4IrMn NPs group showed the highest temperature increase at the thrombosis sites due to their favorable thrombus targeting ability. Moreover, the group treated with 4IrMn NPs demonstrated a superior ability to dissolve a thrombus compared to both PBS and UK. Over time, blood perfusion improved reaching ≈69.3%, with no observed secondary embolism during this process. This success was attributed to the dual‐mode thrombolysis facilitated by PDT/PTT, activated by laser irradiation (Figures 5f,g; Figure S22, Supporting Information). At the end of all the treatments, the carotid arteries were harvested for sectioning. Hematoxylin and Eosin (H&E) staining was performed on the vascular sections (Figure 5h). The thrombosis model exhibited clearly visible emboli within the blood vessels. Notably, the size of these emboli was reduced to varying extents following different treatments. The combination of 4IrMn NPs and irradiation yielded the most effective thrombolytic response, with the smallest thrombotic region observed in the arterial sections. The relative volume of clots in the group treated with 4IrMn NPs decreased by ≈60% (Figure 5i). These findings demonstrated that the synergistic approach of thrombosis ‐targeted PTT / PDT significantly enhanced thrombolytic efficacy.

**Figure 6:**
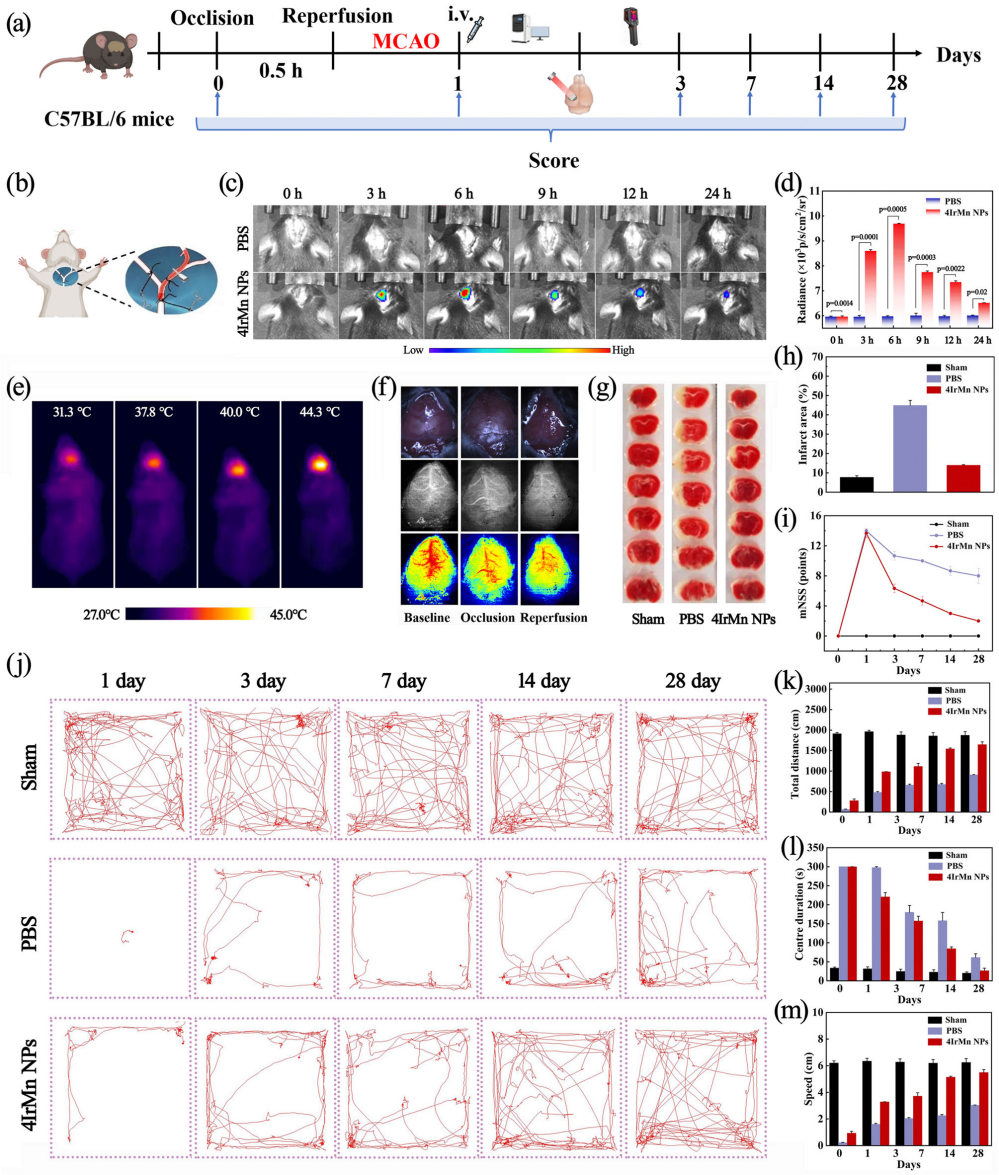
a) Treatment options for ischemic stroke. b) Schematic diagram of surgery for ischemic stroke. c) Representative time‐dependent CL images for PBS and 4IrMn NPs‐administered IS model mice. d) Quantitative average CL intensities for different groups of mice after injection of the 4IrMn NPs. e) Infrared imager recorded temperature changes in the head. f) Representative images of mice, the white‐light image of transparent bone window observation, and laser speckle contrast images for cerebral blood flow in MCAO stroke. g) 2,3,5‐Triphenyltetrazolium chloride (TTC) staining for sham and IS groups. h) Infarct volume for sham and IS groups. i) Neurological scores for sham and IS groups (*N* = 13–15). j) The movement trajectory of the mice for different groups at 1, 3, 7, 14, and 28 days. The total distance k), speed l), and center duration m) of open field test for different groups at 1, 3, 7, 14, and 28 days. Data are presented as mean ± SD (*n* = 3 biological replicates). The data were analyzed by unpaired 2‐tailed Student's t‐test using the GraphPad Prism 7 (d). *p* < 0.05 was considered statistically significant. *p < 0.05, **p < 0.01, and ***p < 0.001 versus control group.

Inspired by the excellent in vitro results, 4IrMn NPs were then tested in vivo using a mouse model of intraluminal middle cerebral artery occlusion (MCAO) (**Figure** **6**a), which simulates ischaemic conditions and real‐time monitoring of early IS processes by NIR‐CL. Laser speckle contrast imaging assessed cerebral blood flow and evaluated the stability of the model (Figure 6f). No CL signal was detected in the brain following intravenous administration, suggesting inadequate penetration of the blood‐brain barrier (BBB). Consequently, 4IrMn NPs were administered directly into the brain, achieving effective perfusion of the brain volume without relying on BBB penetration. However, the efficient penetration of NPs across the BBB is of great significance for stroke treatment efficacy. This necessitates the strategic modification of specific targeting molecules to enhance BBB permeability in future studies. The mice underwent 30 min of cerebral ischemia (Figure 6b), followed by 30 min of reperfusion, after which they were injected with 4IrMn NPs at a concentration of 5 mg kg^−1^. As a control, the sham group was treated with PBS. As shown in Figures 6c,d, the turn‐on CL signal at the ischemia reperfusion injury site was significantly observed, and negligible CL signal was observed in the control sham groups, indicating that the 4IrMn NPs can successfully respond to the endogenic ONOO^−^‐induced NIR‐CL signal in the mouse brain during CIRI. The head temperatures of the mice were recorded using a thermal camera (Figure 6e).

In addition, the cerebral protective effect of 4IrMn NPs was evaluated in vivo and the infarct volumes were observed post‐stroke after treatment. The results of TTC staining and cerebral infarction rate showed that control mice did not show any signs of infarction. After the MCAO operation, the infarction rate was 44.8 ± 2.1%, indicating that the MCAO model was successfully established (Figure 6g). In contrast, when the mice received 4IrMn NPs, the infarct percentage decreased to 13.9 ± 0.35% (Figure 6h), implying significant efficacy in decreasing brain injury. To explore the impact of 4IrMn NPs on long‐term motor and cognitive function recovery of MCAO mice, behavioral tests were performed from pre‐surgery (Pre) to 28 days post‐stroke. To evaluate the effect of 4IrMn NPs on neurological recovery at the indicated time points, the modified neurological severity score (mNSS) with a range of 0−18 is shown. Low mNSS represents a milder neurological deficit and better effective neuroprotective function. High mNSS represents MCAO surgery destroyed the sensorimotor function of mice. Importantly, the score of the 4IrMn NPs‐treated group was significantly lower than that of the PBS‐treated group at all time points, revealing that the 4IrMn NPs possess strong protection against ischemic injury in vivo (Figure 6i).

**Figure 7:**
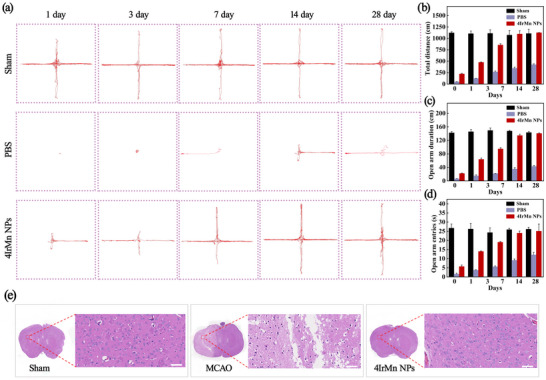
Elevated plus‐maze test in different groups of mice at 1, 3, 7, 14and 28 days. a) The movement trajectory of the mice. b) Total distance. c) Mice entry to open arm duration distance. d) Time spent by mice in the open arm of elevated plus‐maze test in different groups of mice at 1, 3, 7, 14and 28 days. Error bars, mean ± SD (*n* = 3 biological replicates). e) Representative H&E staining results of the brain tissues in the Sham group, MCAO group (PBS), 4IrMn NPs group, respectively. Scale bars = 50 µm.

The behavioral changes were evaluated by open field (Figures 6j–m) and elevated plus‐maze tests (**Figures** **7**a–d). The structure is in the shape of a cross, consisting of two open arms (unobstructed) and two closed arms (with side walls), with a height of 40–55 cm from the ground, simulating the “cliff effect”. The increase in indicators such as the time spent and the number of entries in the open arm by the mice monitors the recovery of their motor nerve function. Compared to the sham groups, the PBS‐treated group exhibits lower total distance and slower moving speed, indicating severely impaired motion due to the stroke. As expected, compared to the PBS‐treated group, larger total distances and faster moving speeds were achieved by the 4IrMn NPs group, showing MCAO mice that had received 4IrMn NPs experienced earlier and sustained improved recovery with significant reversed neurological deficits. Moreover, 4IrMn NPs not only significantly relieved IS in a mouse cerebral ischemia/reperfusion injury model, but also did not cause significant damage to brain tissues (Figure 7e). The 4IrMn NPs treatment for 4 weeks had no effect on the body weight of the mice (Figure S23, Supporting Information). Moreover, the survival rate of the 4IrMn NPs group was higher than that of the PBS‐treated group (Figure S24, Supporting Information).To investigate the cytotoxicity and biosafety aspects, the 4IrMn NPs were further evaluated in vivo by serum biochemical detection and H&E staining of major organs at 3 and 28 days. No significant difference was observed between the untreated mice and the mice injected with 4IrMn NPs regarding the biochemical analysis of their blood (Figure S25, Supporting Information) demonstrating the favorable biocompatibility of the 4IrMn NPs. As presented in Figure S26 (Supporting Information), no pathological changes were found in the major organs of the six groups of mice (namely, with sham for 3 or 28 days; with MCAO + PBS for 3 or 28 days; with MCAO + 4IrMn NPs for 3 or 28 days. Also, negligible cell necrosis was observed in the brains treated with 4IrMn NPs, further indicating a positive therapeutic outcome.

## 3 Conclusion

In summary, Ir and Mn metal cores have been spatially dispersed within a single porphyrin‐based molecule. The obtained 4IrMn NPs overcome steric conflicts between the two metal centers, enabling spatiotemporal control of different functions under endogenous ONOO^−^ or laser activation, including NIR‐CL lesion‐specific imaging, dual‐mode PTT/PDT and neuroprotection, achieving combined coordinated thrombolysis and IS therapy. By systematic consideration of the heavy‐atom effect of cyclometalated Ir(III) cores and the long‐wavelength absorption of porphyrin for superior PTT/PDT performance, together with Mn^2^⁺‐mediated redox activity, the nanosystem achieves three breakthroughs: 1) ONOO^−^‐triggered NIR‐CL enables real‐time localization; 2) PTT/PDT synergistically dissolves thrombi efficiently while avoiding hemorrhagic complications; 3) 4IrMn NPs scavenge cytotoxic RONS (O_2_^−^, ^•^OH) and reduce pro‐inflammatory cytokines (TNF‐α and IL‐6), rescuing ischemic penumbra neurons and limiting infarct volume to 13.9%. These findings validate the therapeutic potential of transition metal‐coordinated porphyrin systems and provide a blueprint for engineering multifunctional nanomaterials against cerebrovascular pathologies.

## 4 Experimental Section

### In Vitro Thrombolytic Efficacy

The artificial thrombus was placed into a 5 mL glass vial, to which the PBS or NPs aqueous solution was added. The mixture was irradiated (1.0 W cm^−2^). The weights of the thrombus before and after thrombolytic treatment were measured to calculate the thrombolysis rate:

(1) $$\begin{array}{ccc} \mathrm{thrombolysis}\;\mathrm{rate} & = & (\mathrm{weight}\;\mathrm{before}\;\mathrm{treatment}-\mathrm{weight}\;\mathrm{after}\;\mathrm{treatment})/\\ & & \mathrm{weight}\;\mathrm{before}\;\mathrm{treatment} \end{array}$$

### In Vitro Cell Cytotoxicity Test

The human umbilical vein endothelial cells (HUVECs) were obtained from Peking Union Medical College Hospital (Peking, China). Fetal bovine serum (FBS), Ham's F12K, heparin, endothelial cell growth supplement (ECGS), L‐glutamine, penicillin, and streptomycin were obtained from Corning (New York, USA). The cells were maintained in Ham's F12K medium with heparin (0.1 mg mL^−1^), ECGS (0.05 mg mL^−1^), 10% FBS, 1% L‐glutamine, and 1% penicillin/streptomycin at 37 °C containing 5% CO_2_. For in vitro cytotoxicity tests, HUVECs were seeded into 96‐well plates with a density of 1 × 10^4^ per well. After 24 h, the cells were incubated with NPs for another 24 h. The MTT assay was conducted following the standard protocol.

The mouse hippocampal neurons (HT22) cells were regularly checked for mycoplasma contamination. HT22 cells were cultured in Dulbecco's Modified Eagle's Medium (DMEM) (Gibco, Grand Island, NY, USA) supplemented with 10% fetal bovine serum (FBS) (Gibco), penicillin (100 U mL^−1^), and streptomycin (100 µg mL^−1^), and the cultures were maintained at 37 °C in a humidified atmosphere containing 5% CO_2_. Cells were divided into three groups for treatment with H_2_O_2_ (200 µm): i) control group; ii) HT22 cells were incubated with H_2_O_2_ alone for 24 h; and iii) HT22 cells were incubated with NPs for 24 h and then treated with H_2_O_2_ for another 24 h.

### Animal Experiments

All animal experiments were approved by the Ethics Committee for Animal Experimentation of China Technology Industry Holdings (Shenzhen) Co., Ltd (Ethics approval number: 20 240 030). All procedures complied with the Animal Research: Reporting In Vivo Experiments (ARRIVE) guidelines. The male C57 BL/6J mice (8–10 weeks old) were housed in a pathogen‐free environment with no more than three mice per cage. They were subjected to a 12‐hour dark‐light cycle and were given unrestricted access to food and water.

### Statistical Analysis

Data were denoted as the mean ± standard deviation (SD). The significance between experimental and control groups was determined by unpaired 2‐tailed Student's t‐test using the GraphPad Prism 7. A value of *p* < 0.05 was considered statistically significant. *p < 0.05, **p < 0.01, and ***p < 0.001.

## Conflict of Interest

The authors declare no conflict of interest.

## Supporting information



Supporting Information

## Data Availability

The data that support the findings of this study are available in the supplementary material of this article.
