# Supplementary material for: A Metalloporphyrin Nanosystem Enables Non‐Invasive Visualization and Specific Treatment for Thrombosis and Ischemic Stroke
Source: Adv Sci (Weinh). 2025 Oct 5;12(47):e15079. doi: 10.1002/advs.202515079 (PMC12713076; doi:10.1002/advs.202515079)
Supplement: Supplementary file 1 — Supporting Information [file ADVS-12-e15079-s001.pdf]

Supporting Information

A Metalloporphyrin Nanosystem Enables Non-Invasive Visualization and Specific Treatment for Thrombosis and Ischemic Stroke

Ziwei Wang, Liping Zhang, Zihan Wu, Nan Xiao, Wenxin Zheng, Yachao Wang, Geling Xu, Dongxia Zhu*, Martin R. Bryce*, Lijie Ren* and Ben Zhong Tang**

General Materials and Methods.

Materials for organic synthesis were purchased from Energy Chemical Company. 1,2-distearoyl-sn-glycero-3-phosphoethanolamine-*N*-[maleimide(poly(ethyleneglycol))-2000] (DSPE-PEG-MAL) was purchased from Laysan Bio, Inc. (Arab, AL). c(RGDfC) was supplied by Hangzhou Special Peptide Biotechnology Co. 1,3-Diphenylisobenzofuran (DPBF) were purchased from Energy Chemical Company. RPMI Medium 1640 was purchased from Solarbio Life Science Company. Fetal bovine serum (FBS) was purchased from Sigma-Aldrich. 3-(4,5-dimethyl-2-thiazolyl)-2,5-diphenyl-2*H*-tetrazolium bromide (MTT), 7'-dichlorofluorescence diacetate (DCFH-DA) and the cell viability (live dead cell staining) assay kit were purchased from Shanghai Beyotime Biotechnology Co. Ltd.

^{1}H NMR spectra were recorded at 25 °C on a Varian 600 MHz spectrometer. Mass spectra were recorded on a Bruker autoFlex III instrument. UV-vis absorption spectra were recorded on a Shimadzu UV-3100 spectrophotometer. The photoluminescence spectra were recorded on an Edinburgh FLS920 spectrofluorimeter under air at room temperature. FTIR spectra were obtained on a NICOLET iS50 instrument. Transmission electron microscopy (TEM) images of the samples were taken by a TECNAI F20 microscope. Diameter and diameter distribution of the nanoparticles were determined by a Malvern Zetasizer Nano instrument for dynamic light scattering (DLS).

Confocal laser scanning microscopy (CLSM) images were taken using a LSM 800 Zeiss, Germany.

Preparation of NPs.

The nanoparticles were prepared by a modified nanoprecipitation method. The synthesis process was carried out at 25 °C. 4IrMn (1 mg) and DSPE-PEG-MAL (2 mg) were dispersed in 1 mL of THF. After the compounds were completely dissolved, the above stock solution was added dropwise to 10 mL of purified water under vigorous stirring. The mixture was stirred overnight in a fume hood to evaporate the organic solvent. Then, c(RGDfC) was dispersed in 1 mL of water and the peptide was loaded by thiol-Michael addition reaction for 16 h at room temperature. The residual c(RGDfC) peptide was then removed using a dialysis bag. The obtained 4IrMn NPs were stored in a refrigerator at 4 °C for subsequent experiments. TPP NPs, IrNPs, IrMn NPs and 4Ir NPs were prepared in the same way.

In Vitro Photothermal Measurements.

To investigate the photothermal performance of NPs, different NPs aqueous dispersions (1 mL) were irradiated with 635 nm laser (0.8 W cm^{-2}) for 5 min, and the temperature change was recorded by a thermocouple, and the photothermal images were acquired by an infrared thermal imager. In addition, the photothermal effect of nanoparticles was investigated using different laser power intensities (0.2, 0.4, 0.6 and 0.8 W cm^{-2}) and the temperature change of different concentrations of NPs within 5 min were measured under the same power of laser irradiation. In order to study the NPs' photostability, five cycles of laser on/off were carried out.

Photothermal Conversion Efficiency of the 4IrMn NPs.

The photothermal conversion efficiency of the 4IrMn NPs was calculated according to a reported method.[1] First, the 4IrMn NPs (4×10^{-5} M) in aqueous solution were irradiated with 635 nm laser (0.8 W cm^{-2}) for 300 s and then the laser was turned off.

After about 800 s, the solution was cooled to room temperature. The photothermal conversion efficiency (η) was calculated according to the following equation

$$\eta=[hA(T_{Max}-T_{surr})-Q_{dis}]/I(1-10^{-A}) \quad (1)$$

where h and A respectively represent the heat transfer coefficient and the surface area of the container, T_{Max} and T_{surr} represent the maximum temperature and the room temperature of the environment, Q_{dis} represents the heat dissipation of the solvent (water), I is the laser power employed (0.8 W cm^{-2}), and A is the absorbance of 4IrMn NPs at 635 nm. The value of hA is calculated from the following equation

$$\tau_s=m_Dc_D/hA \quad (2)$$

where τ_s is the time constant for heat transfer of the system, which was determined to be τ_s = 128.99 from Figure S11; m_D and c_D are the mass and heat capacity, respectively, of the deionized water used to disperse the NPs. Q_{dis} represents the heat dissipation from the laser absorbed by the water, so Qdis was calculated according to the following equation

$$Q_{dis}=m_Dc_D(T_{Maxwater}-T_{surr})/\tau_{swater} \quad (3)$$

where $T_{max\ water}$ is the highest temperature of water and $\tau_{s\ water}$ is 167.8; thus, Q_{dis} was calculated to be 0.537 W. According to the data obtained and Equation (1), the photothermal conversion efficiency of the 4IrMn NPs was 57.5%.

$^1O_2$ quantum yield measurements

The $^1O_2$ quantum yield of 4IrMn NPs in water (Φ) upon laser irradiation (635 nm, 0.8 W cm^{-2}) was determined using 9,10-anthracenediyl-bis(methylene) dimalonic acid

(ABDA) as an indicator and using Rose Bengal (RB) as the standard reference. ABDA solid (200 μM) was dissolved in DI water. The 4IrMn NPs (1.5×10^{-5} M) or RB (6 μg mL^{-1}) was then added in aqueous solution. The absorbance decrease of ABDA at 400 nm was recorded for different durations of irradiation to obtain the decay rate of the photosensitizing process. The $^1O_2$ yield was calculated using the following equation:

$$\Phi_{4IrMn\ NPs} = \Phi_{RB}\ (K_{4IrMn\ NPs} \times A_{RB}) / K_{RB} \times A_{4IrMn\ NPs}$$

where $K_{4IrMn\ NPs}$ and K_{RB} are the decomposition rate constants of the photosensitizing process determined by the plot ln (A_0/A) versus irradiation time. A_0 is the initial absorbance of ABDA while A is the ABDA absorbance after different irradiation times. $A_{4IrMn\ NPs}$ and A_{RB} represent the light absorbed by NPs and RB, which are determined by integration of the absorption bands in the wavelength range of 400-800 nm.

Preparation of Reactive Oxygen and Nitrogen Species (RONS) Solutions.

The RONS solutions containing H_2O_2, TBHP and ClO^-, respectively, were purchased and diluted to the experimental concentration (200 μM) by 1×PBS (pH 7.4). In brief, •OH was produced by addition of ferrous chloride (0.1 M, 1 mL) into H_2O_2 solution (1.0 M, 1 mL) through a Fenton reaction. Accordingly, the concentration of •OH is the same as that of Fe^{2+} (50 mM). $O_2^{\bullet-}$ was generated from KO_2 (35.5 mg), which was directly added into dimethyl sulfoxide (10 mL) at a final concentration of 50 mM. $ONOO^-$ was prepared by addition of sodium hydroxide (1.5 M) into mixtures of sodium nitrite (0.6 M), hydrogen peroxide (0.7 M) and hydrochloric acid (0.6 M) at 0 °C, followed by purification through a short column of manganese dioxide to remove excess hydrogen peroxide. The concentration of $ONOO^-$ was determined by measurement of the absorption at 302 nm. $C[ONOO^-] = Abs_{302\ nm}/1.67$ (mM). $^1O_2$ was produced by addition of ClO^- solution (100 mM, 1 mL) into H_2O_2 solution (200 mM, 1 mL). According to this reaction, the concentration of $^1O_2$ is the same to that of ClO^- (50 mM). NO_2^- was generated from $NaNO_2$ (35 mg), which was directly added into deionized water (10 mL) at a final concentration of 50 mM.

Chemiluminescence and Fluorescence Imaging In Vitro.

For in vitro chemiluminescence imaging, including determinations of RONS selectivity, chemiluminescence spectra, and $ONOO^{-}$-activated sensitivity, etc., the concentration of 4IrMn NPs was 200 μg mL^{-1}. The chemiluminescence imaging was carried out with an Xenogen IVIS Lumina II system in a bioluminescent mode (exposure time 60 s) post RONS (200 μM) addition, unless otherwise specified. Tests were every 20 nm/step by the different filters. The environmental temperature for in vitro afterglow imaging was kept at 37 °C. The fluorescence spectra of 4IrMn NPs were measured by the same IVIS instrument in a fluorescent mode with excitation at 465 ± 10 nm (exposure time 1 s). The fluorescence and chemiluminescence images were analyzed by ROI analysis using the Living Image 4.2 Software.

FITC-Labeled Fibrin Clots Assay.

FITC-labeled fibrin clots were induced by the addition of 10 U mL^{-1} thrombin and 2.5 mM $CaCl_2$ into a fibrinogen solution containing 1 mg mL^{-1} fibrinogen (200 μL) and 1 mg mL^{-1} FITC-labeled fibrinogen (20 μL), followed by incubation at 37 °C for 1 h. The clot was incubated with 4IrMn NPs (100 μg mL^{-1}, 600 μL) and further irradiated with a 635 nm laser (0.8 W cm^{-2}, 2 min) and evaluated through a CLSM.

In vitro hemolysis experiment.

A hemolysis assay was conducted to evaluate the hemocompatibility of 4IrMn NPs. Primarily, the whole blood of a mouse treated with citrate was gathered and centrifuged at 3000 rpm for 3 min to obtain erythrocytes. Then, the erythrocytes were resuspended in an equal volume of PBS. PBS or water solution was used as negative and positive controls, respectively, which were added to 2 mL microtubes containing erythrocytes. Various concentrations (5, 10, 50, 100, 150 and 200 μg mL^{-1}) of 4IrMn NPs were incorporated into the erythrocytes for comparison with the controls. The mixture was then incubated in a 37 °C water bath for 2 h, followed by centrifugation at 3000 rpm for 10 min. After centrifugation, 100 μL of supernatant from each sample was transferred to a 96-well plate and the absorbance was recorded with an enzyme

marker at 540 nm. The equation for calculation of hemolysis rate (%) is presented below. Hemolysis rate (%) = ($A_0 - A_1$) × 100%/ ($A_2 - A_1$).

In vitro thrombus targeting of 4IrMn NPs.

Fresh blood was collected and divided into tubes with equal volumes (5 mL). Each tube was mixed with thrombin (5 U μL^{-1}) and $CaCl_2$ (3 mM) to induce the formation of clots which were cut into equal sizes. The artificial thrombus was then respectively incubated with PBS, 4IrMn [no c(RGDfC] or 4IrMn NPs aqueous solution (0.1 mg mL^{-1}) to verify the targeting ability of the NPs. After incubation for 2 h, 4 h or 6 h, the thrombus clots were taken out and washed three times with PBS. The chemiluminescence of the thrombus clots was analyzed using the IVIS imaging system.

In vitro thrombolytic efficacy

The artificial thrombus was placed into a 5 mL glass vial, to which the mixture of 2.5 mL of PBS and 0.5 mL of different NPs solutions was added. The mixture was irradiated with a 635 nm laser. The weights of thrombus before and after thrombolytic treatment were measured to calculate the thrombolysis rate according to the equation: thrombolysis rate = (weight before treatment – weight after treatment)/weight before treatment. In addition, the treated thrombus clots were sectioned and stained with hematoxylin and eosin (H&E) to evaluate the thrombolysis efficiency.

Cell Culture.

The mouse hippocampal neuron (HT22) cells were regularly checked for mycoplasma contamination. HT22 cells were cultured in Dulbecco's Modified Eagle's Medium (DMEM) (Gibco®, Grand Island, NY, USA) supplemented with 10% fetal bovine serum (FBS) (Gibco®), penicillin (100 U mL^{-1}), and streptomycin (100 µg mL^{-1}) and the cultures were maintained at 37 °C in a humidified atmosphere containing 5% CO_2. Cells were divided into three groups for treatment with H_2O_2 (200 µM): (i) control group; (ii) HT22 cells were incubated with H_2O_2 alone for 24 h; and (iii) HT22 cells were incubated with NPs for 24 h and then treated with H_2O_2 for another 24 h.

In Vitro Cell Cytotoxicity Test.

The human umbilical vein endothelial cells (HUVECs) were obtained from Peking Union Medical College Hospital (Peking, China). Fetal bovine serum (FBS), Ham's F12K, heparin, endothelial cell growth supplement (ECGS), L-glutamine, penicillin, and streptomycin were obtained from Corning (New York, USA). The cells were maintained in Ham's F12K medium with heparin (0.1 mg mL^{-1}), ECGS (0.05 mg mL^{-1}), 10% FBS, 1% L-glutamine and 1% penicillin/streptomycin at 37 °C containing 5% CO_2. For in vitro cytotoxicity tests, HUVECs were seeded into 96-well plates with a density of 1×10^4 per well. After 24 h, the cells were incubated with 4IrMn NPs for another 24 h. The MTT assay was conducted following the standard protocol.

In Vitro Intracellular ROS and RONS Evaluation.

The reactive oxygen species (ROS) and RONS were imaged using specific agents, namely, 2'7'-dichlorofluorescein diacetate (DCFH-DA) for ROS, dihydroethidium (DHE) for $O_2^{\bullet-}$, hydroxyphenyl fluorescein (HPF) for •OH. HUVECs (1×10^5 cells/well) were pretreated with four concentrations of 4IrMn NPs for 24 h, washed with phosphate buffered saline (PBS) three times, and then were incubated with DCFH-DA (10 μM), DHE (10 μM) and HPF (5 μM) for 30 min at 37 °C. Subsequently, residual agents were removed using PBS. The images were obtained using a confocal microscope (LSM 800 Zeiss, Germany). Each experiment was performed independently three times.

Construction of thrombus model

The male C57 BL/6J mice (8-10 weeks old) were anesthetized using isoflurane (2% of isoflurane, 0.5 mL min^{-1} oxygen) and the neck hair was shaved off. Then the skin around the neck was cut with surgical scissors, and the connective tissue and fat were peeled away to expose the carotid vessels. A 10% aqueous solution of $FeCl_3$-soaked filter paper (2 × 1 mm) was placed on the surface of the exposed carotid artery for 10 min, then the filter paper was removed. The vessel and surrounding tissue were washed

with sterilized saline solution. An aggregated embolus could be observed under the somatic microscope. A laser speckle imaging system (RWD, RFLSI III, Shenzhen, China) was used to monitor the hemodynamic changes before and after the induction of carotid artery thrombus using $FeCl_3$. All animal procedures were approved by the China Technology Industry Holdings (Shenzhen) Co., Ltd.

In vivo Chemiluminescence imaging of thrombus

The C57 BL/6J mice (8-10 weeks old) with thrombus model were intravenously treated with PBS or 4IrMn NPs (5 mg kg^{-1}), respectively. The mice were then anesthetized using isoflurane (2% of isoflurane, 0.5 mL min^{-1} oxygen), and placed on a heating platform at 37 °C. At predetermined time points (0, 30, 60, 90, 120, 180, and 240 min) post NPs administration, the in vivo chemiluminescence imaging was carried out using an IVIS imaging system.

MCAO Model

The middle cerebral artery occlusion (MCAO) model was established using the suture method. Mice were first anesthetized with 5% isoflurane and maintained under 1.5% isoflurane after deep anesthesia. Then the neck skin of the mice was cut off and a silicon suture (Xinong Technology) was inserted from the right common carotid artery (CCA) into the internal carotid artery (ICA) at a depth of 8–10 mm. The successful construction of the MCAO model was confirmed by the laser speckle contrast imaging system (RFLSI III, RWD) with a more than 80% decrease in regional cerebral blood flow (rCBF). After 60 min, reperfusion was achieved after the removal of the silicon suture. The procedure for the sham-operated group was similar to the model group except for the insertion of the silicon suture. Mice established with the MCAO model were randomly divided into 2 groups (n = 3–10) and separately intravenously injected with 100 μL of saline and 4IrMn NPs post-stroke. In vivo investigations excluding long-term evaluations were performed on day 3 post-stroke.

Laser Speckle Contrast Imaging.

Mice were anesthetized with 1-1.5% isoflurane. The hair was shaved, the skull was exposed, and cerebral blood flow (CBF) was detected using a laser speckle contrast imaging system (RWD, Shenzhen, China). The images and videos were analyzed using RWD software.

Drug-delivery process.

Pre-irradiated 4IrMn NPs (5 mg mL^{-1}, 10 μL) or saline (10 μL) preparation were administered into the striatum (+2.0 mm anterior/posterior, +1.0 mm medial/lateral, and +2.0/2.5 mm dorsal/ventral relative to the bregma) using a stereotaxic device (RWD, Life Science, Shenzhen, China) and a Hamilton syringe (10 μL Hamilton, Cat. No.80300 USA).

Infarct Volume.

Mice were killed 72 h after MCAO (n = 10). The brains were harvested and 2,3,5-triphenyltetrazolium chloride (TTC) was used to stain the brain slices. In the survival tissue, TTC was a red formazan product, while the infarct areas were pale. The infarct area of each section (1 mm) was calculated by subtracting the non-infarcted area in the ipsilateral hemisphere from the total area of the contralateral hemisphere. Lastly, the total infarct volume was obtained by summing the infarct areas in all sections and multiplying by the section thickness. The quantification analysis was conducted by software ImageJ to obtain the infarct area of the coronal section.

Neurological Scores.

The modified neurological severity scores (mNSS) test, consisting of a set of tests, was used to evaluate many aspects of neurological functions, including motor function and sensory functional reflexes. (A translocator protein 18 kDa agonist protects against cerebral ischemia/reperfusion injury). The score was graded from 0 to 18. One point was given if a mouse failed to perform a test. A higher score indicates more severe impairment. The rating scale was as follows: a score of 13-18 indicates severe injury; 7-12 indicates moderate injury; 1-6 indicates mild injury. Two investigators who were

unaware of the mouse groups performed the behavior tests to evaluate the neurological deficits at 1, 3, 7, 14 and 28 days after MCAO.

Open Field Test.

The open field test consisted of a square field (30 cm), with the floor divided into twelve parts. For a 5-min period, each animal was placed in the center of the arena, and the number of lines it crossed with its four paws were recorded. All tests were taped with a video recorder. After each test, the apparatus was carefully cleaned with 10% ethanol solution.

Elevated Plus Maze Test.

The elevated plus-maze apparatus consisted of two open (30 × 5 cm) and two enclosed (30 × 5 × 25 cm) arms, with a central platform (5 × 5 cm). The open arms were surrounded by lateral bars (0.25 cm in height) to prevent the animal from falling. The apparatus was raised 38.5 cm above ground level. Each animal was placed on the central platform facing one of the enclosed arms. For 5 min, frequencies of entries into either open or enclosed arms and the time spent in each arm type were recorded (in seconds). The number of entries into the enclosed arms was used as an index of general activity. All tests were taped with a video recorder. After each test, the apparatus was carefully cleaned with 10% ethanol solution.

In vivo Biosafety Evaluation.

To test the potential toxicity of 4IrMn NPs, healthy C57BL/6 mice (8-10 weeks old) were intravenously injected with PBS or 4IrMn NPs (5 mg kg^{-1}). One week after PBS or NPs administration, the mice were euthanized, and blood was drawn. Then the hematological parameters, including white blood cell count, red blood cell count, hemoglobin concentration, platelet crit were analyzed using an automated hematology analyzer.

Statistical Analysis

Data are denoted as the mean ± standard deviation (SD). The significance between experimental and control groups was determined by unpaired 2-tailed Student's t-test using the GraphPad Prism 7. A value of $p < 0.05$ was considered statistically significant. *$p < 0.05$, **$p < 0.01$, ***$p < 0.001$.

In Vivo Side Toxicity Evaluation.

The mice were sacrificed at days 3 and 28, and major organs and the brain were collected and fixed in 4% paraformaldehyde. Then they were embedded into paraffin and sliced at a thickness of 5 μm. Slices were stained with hematoxylin and eosin (H&E) and imaged by optical microscopy.

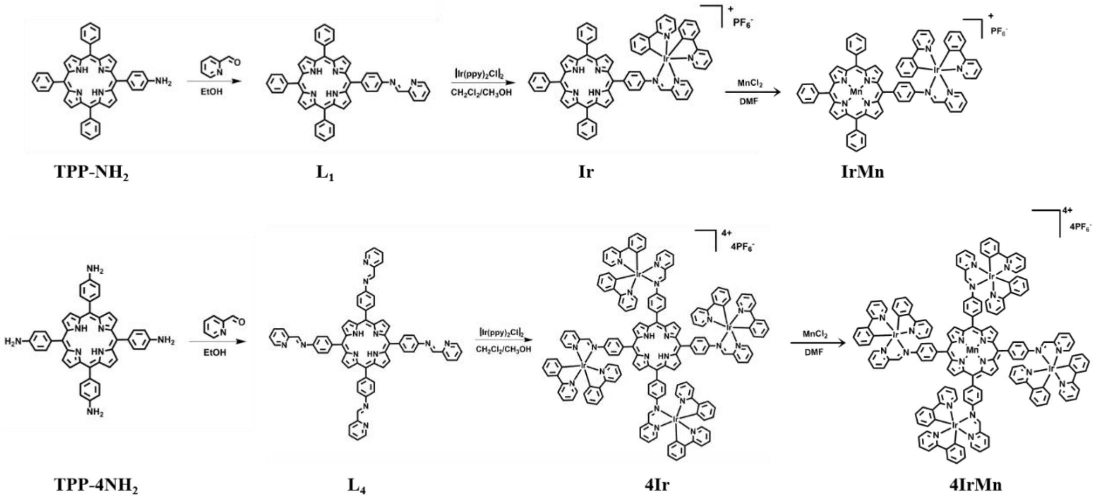

Scheme S1. The synthetic routes to IrMn and 4IrMn.

Synthesis of IrMn and 4IrMn.

Ir and 4Ir were synthesized on the basis of previous work.[2] Ir (0.1 mmol, 0.1219 g)/4Ir (0.1 mmol, 0.3034 g) and $MnCl_2$ (0.5 mmol, 0.062 g) were added to a 50 mL burette with DMF (25 mL) as the reaction solvent, and the mixture was stirred at 80 °C for 8 h. The product was extracted with dichloromethane and water, and the solvent was removed by rotary evaporation, and the red solid product, i.e., **IrMn**/**4IrMn** was dried and obtained in a yield of 80%/82%: molecular formula $C_{72}H_{48}IrMnN_8$/$C_{156}H_{108}Ir_4MnN_{20}$. HRMS (ESI) *m*/*z*:$[M]^+$ calcd for $C_{72}H_{48}IrMnN_8$, 1272.38; found, 1272.30 g/mol. HRMS (ESI) *m*/*z*: $[M]^+$ calcd for

$[C_{156}H_{108}Ir_4MnN_{20}]2CN$, 3138.4; found, 3138.4 g/mol.

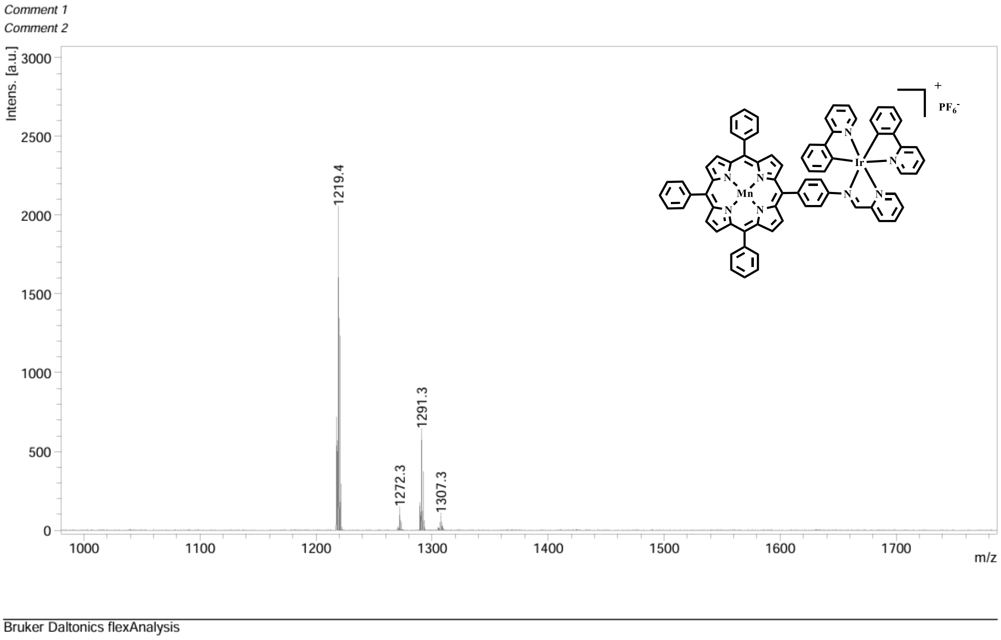

Figure S1. Mass spectrum of IrMn at room temperature.

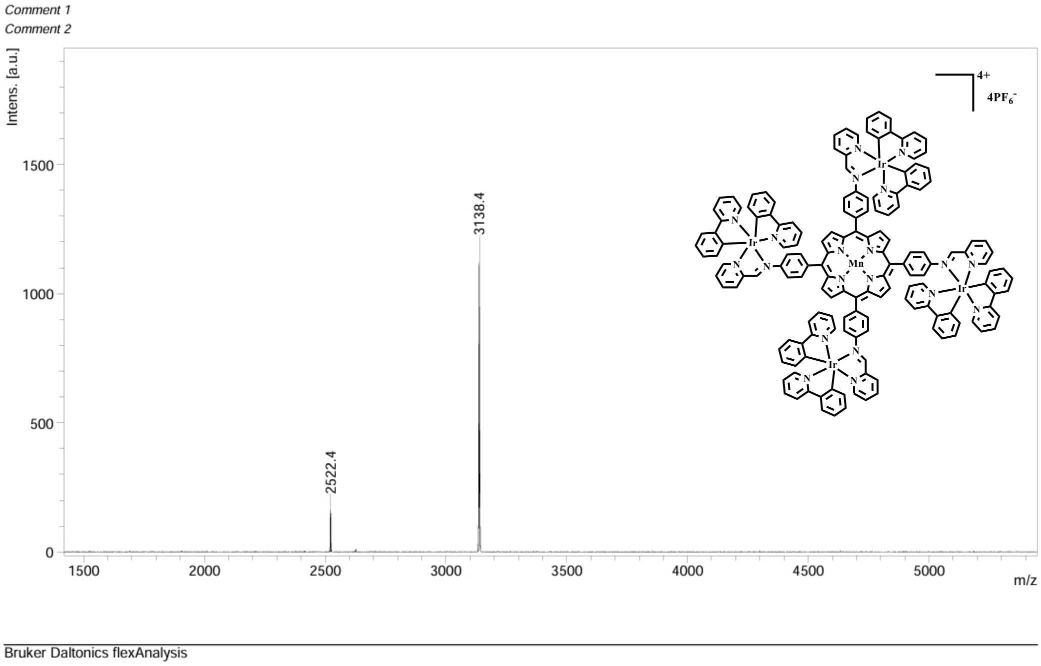

Figure S2. Mass spectrum of 4IrMn at room temperature.

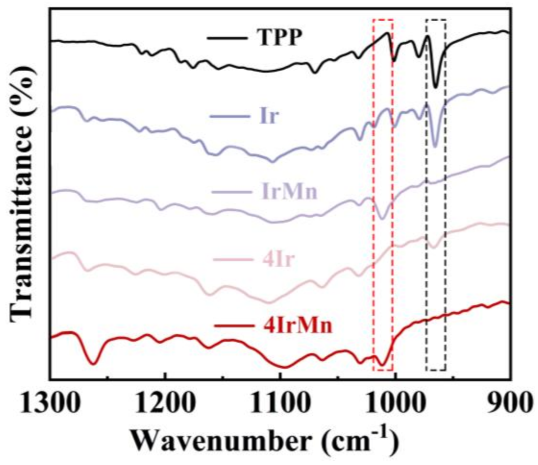

Figure S3. FT-IR spectra of TPP, Ir, IrMn, 4Ir and 4IrMn.

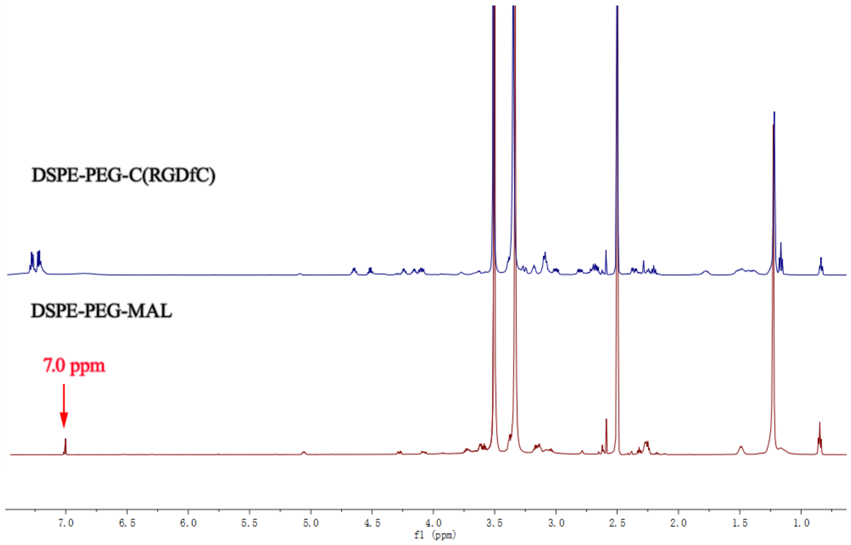

Figure S4. ^{1}H NMR spectra of DSPE-PEG-MAL and DSPE-PEG-c(RGDfC) (600 MHz, DMSO-d_6). The disappearance of the maleimide group signal at 7.0 ppm indicated the successful conjugation of c(RGDfC) to DSPE-PEG-MAL.

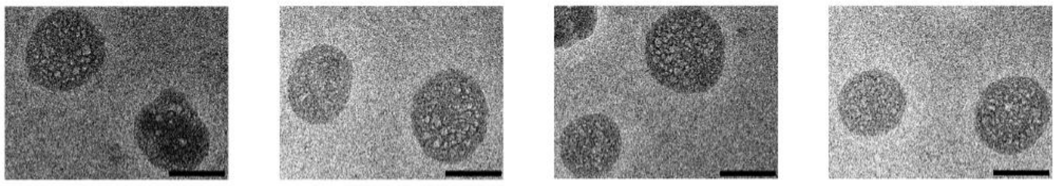

Figure S5. TEM images from left to right, in sequence: TPP NPs, Ir NPs, IrMn NPs and 4Ir NPs. (Scale bars = 100 nm)

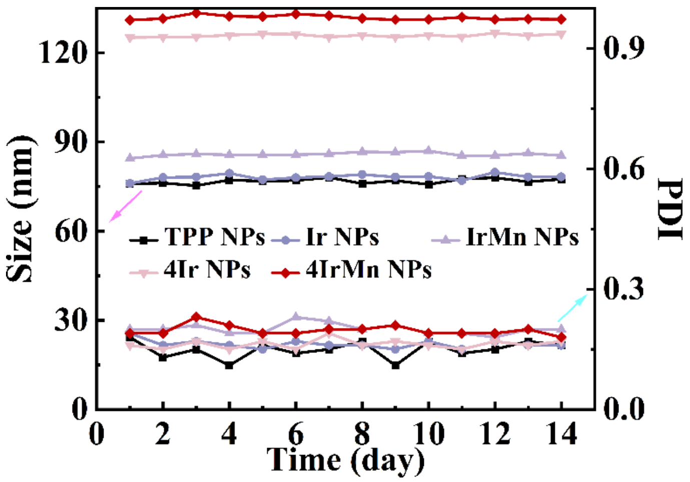

Figure S6. Stability of size distribution changes over 14 days for TPP NPs, Ir NPs, IrMn NPs, 4Ir NPs and 4IrMn NPs.

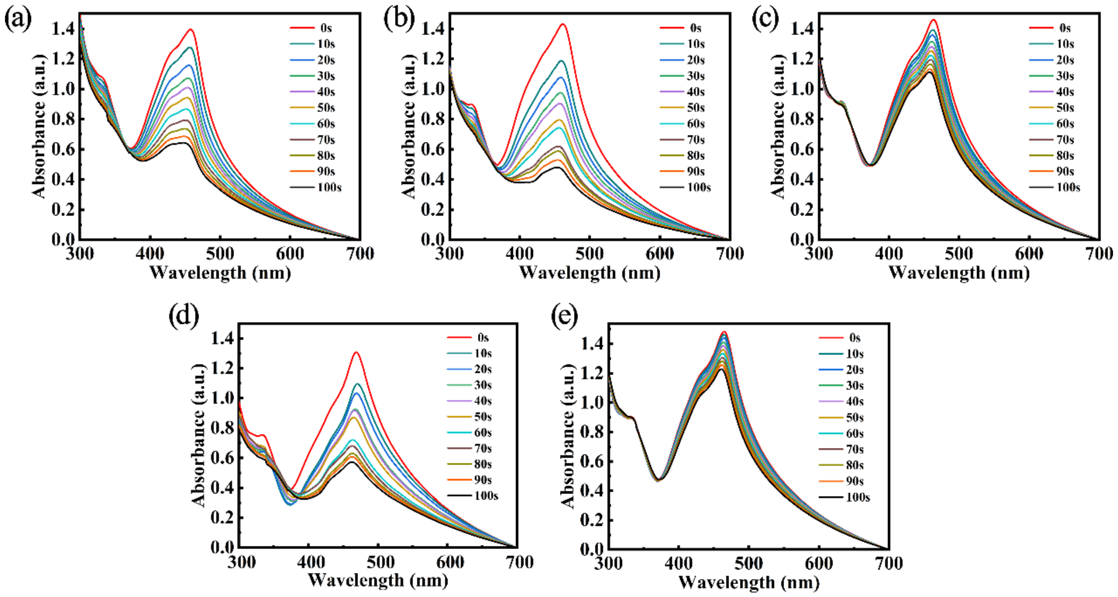

Figure S7. (a-e) UV–vis absorption spectral changes of DPBF (1.5×10^{-5} M in water) in the presence of 4IrMn NPs, 4Ir NPs, IrMn NPs, Ir NPs and TPP NPs upon exposure to light (635 nm, 0.8 W cm^{-2}) for 100 s.

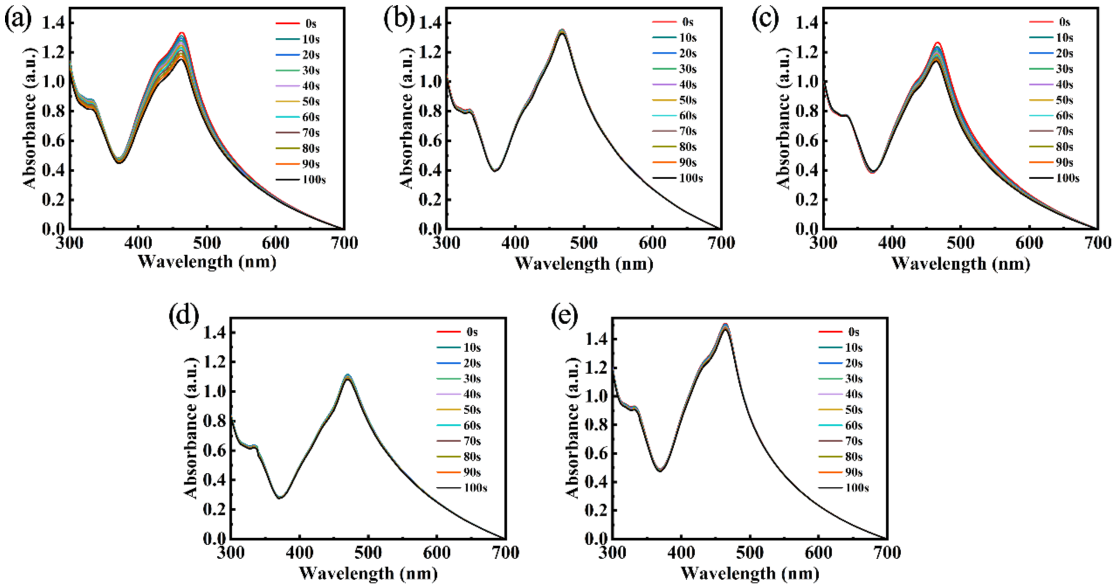

Figure S8. (a-e) UV–vis absorption spectral changes of DPBF (1.5×10^{-5} M in water) in the presence of 4IrMn NPs, 4Ir NPs, IrMn NPs, Ir NPs and TPP NPs for 100 s.

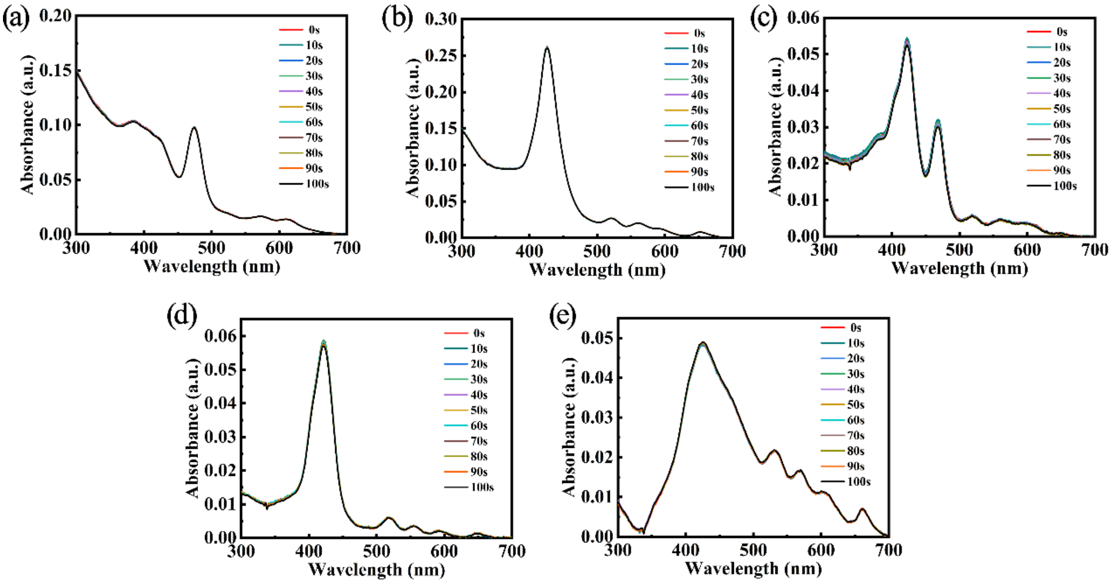

Figure S9. (a-e) UV–vis absorption spectral changes of 4IrMn NPs, 4Ir NPs, IrMn NPs, Ir NPs and TPP NPs in water upon exposure to light (635 nm, 0.8 W cm^{-2}) for 100 s.

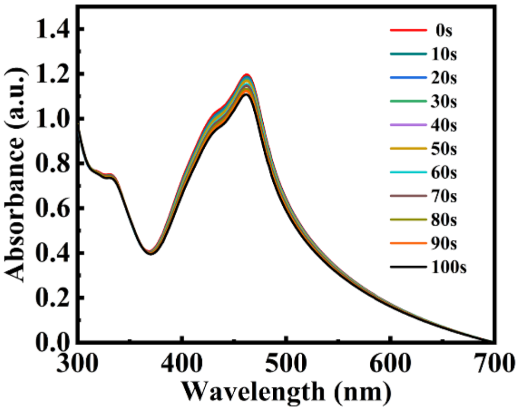

Figure S10. UV–vis absorption spectral changes of DPBF (1.5×10^{-5} M in water) upon exposure to light (635 nm, 0.8 W cm^{-2}) for 100 s.

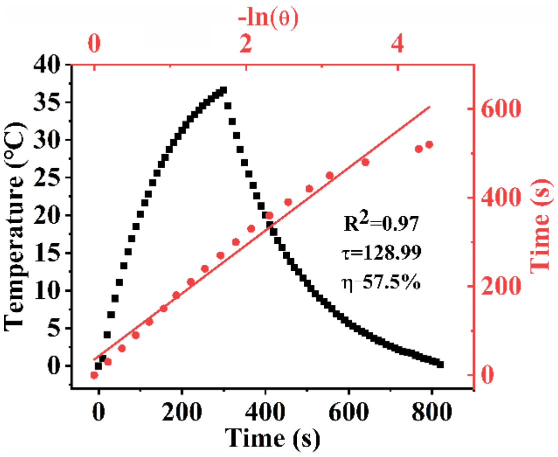

Figure S11. The photothermal conversion efficiency of 4IrMn NPs. A plot of cooling time versus negative natural logarithm of the temperature obtained from the cooling stage.

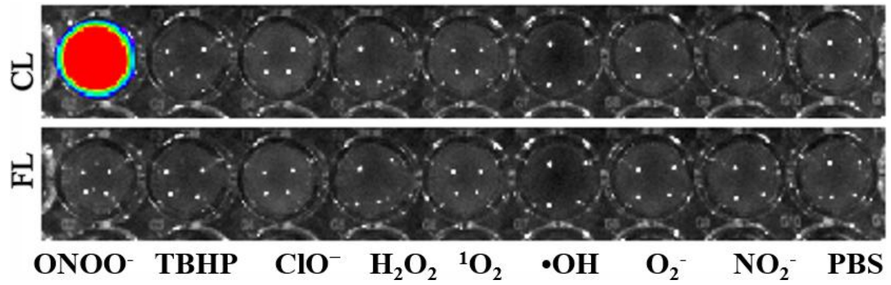

Figure S12. CL and PL imaging of the 4IrMn NPs toward different RONS (200 μM) treatments.

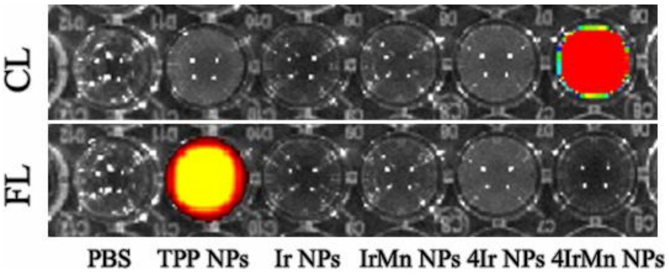

Figure S13. CL and PL images of PBS, TPP NPs, Ir NPs, IrMn NPs, 4Ir NPs and 4IrMn NPs.

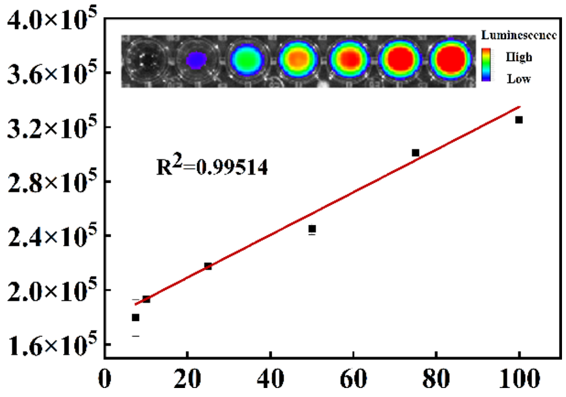

Figure S14. Correlation of persistent CL intensity of 4IrMn NPs versus $ONOO^-$ concentration. Insets: the corresponding CL images acquired on an IVIS imaging system.

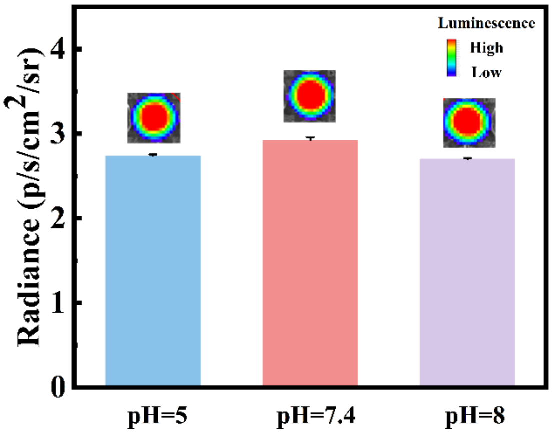

Figure S15. Normalized chemiluminescence plots of 4IrMn NPs at different pH values.

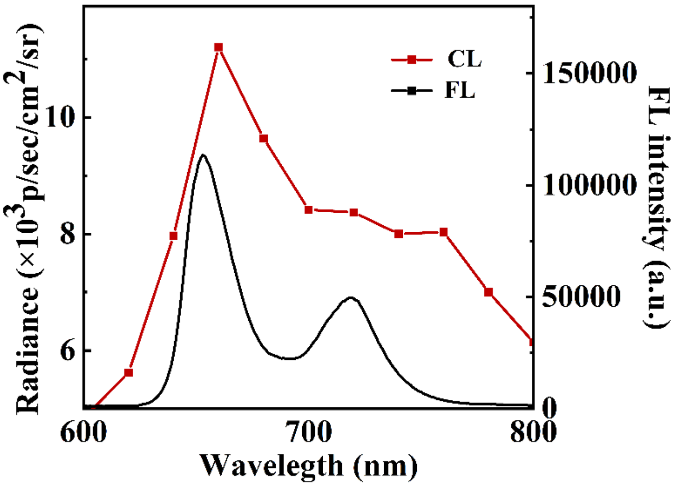

Figure S16. CL and FL spectra of the intermediates of 4IrMn NPs reacted with $ONOO^-$.

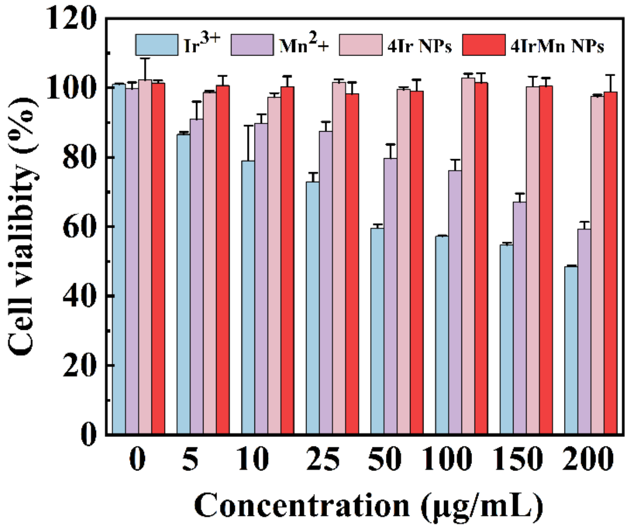

Figure S17. Cell survival rates at different concentrations of free Ir/Mn ions, 4Ir NPs and 4IrMn NPs.

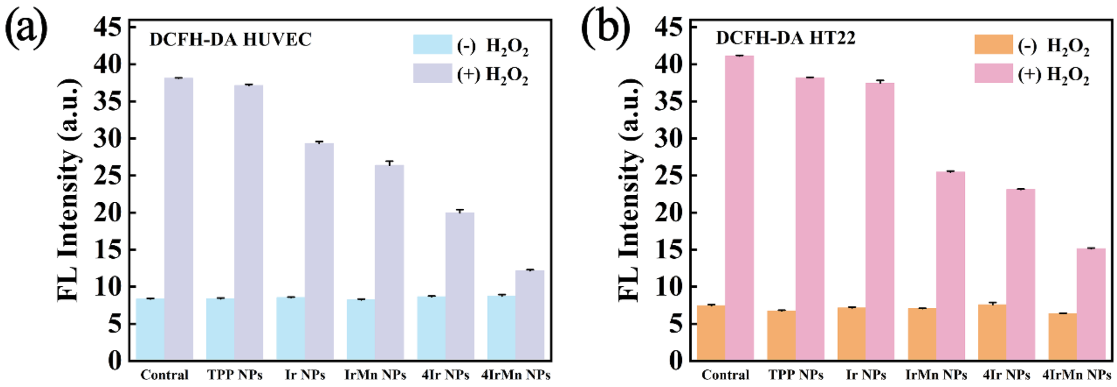

Figure S18. Quantitative analysis of the intracellular fluorescence intensity of NPs scavenged RONS activity induced by H_2O_2-treated HUVEC and HT22 cells in vitro.

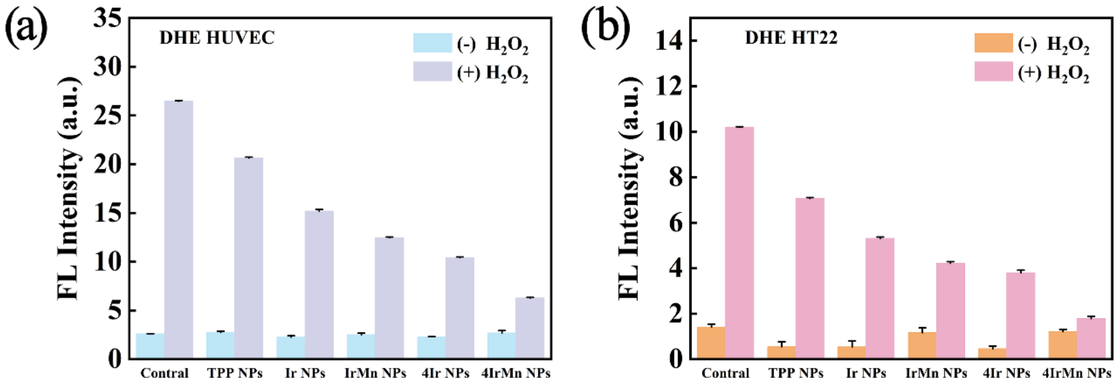

Figure S19. Quantitative analysis of the intracellular fluorescence intensity of NPs scavenged $O_2^{\bullet-}$ activity induced by H_2O_2-treated HUVEC and HT22 cells in vitro.

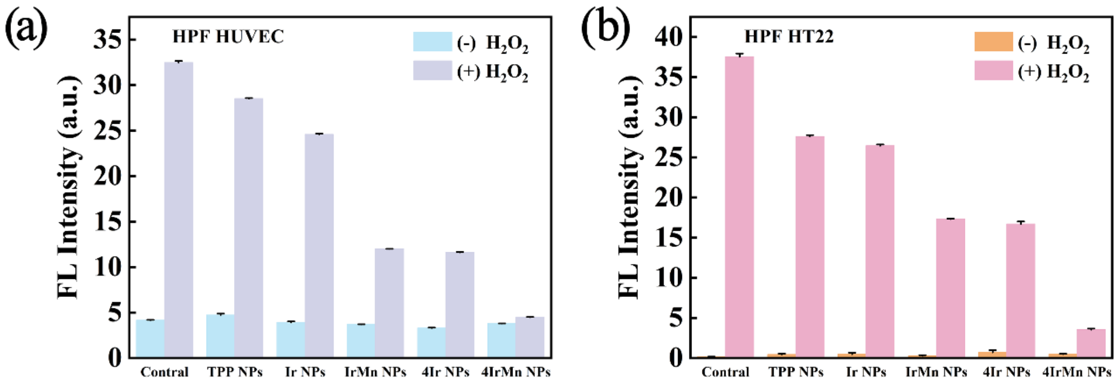

Figure S20. Quantitative analysis of the intracellular fluorescence intensity of NPs scavenged $^{\bullet}OH$ activity induced by H_2O_2-treated HUVEC and HT22 cells in vitro.

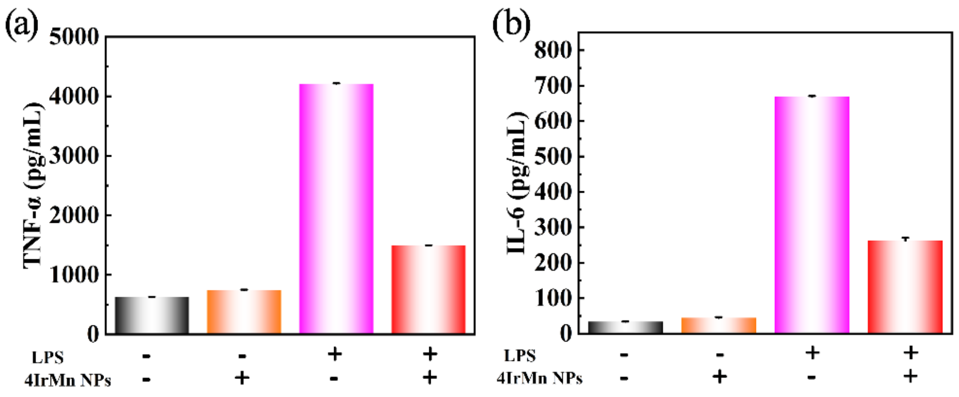

Figure S21. Concentrations of (a) TNF-α and (b) IL-6 were determined by an enzyme-linked immunosorbent assay (ELISA).

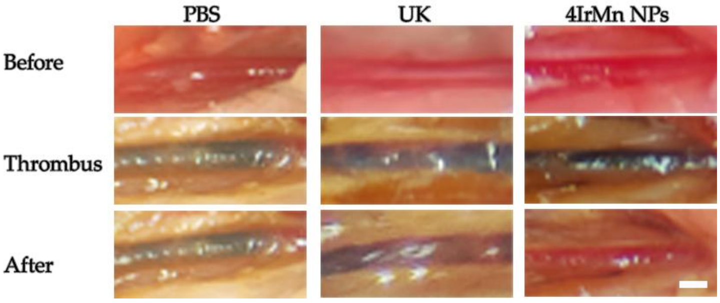

Figure S22. Representative thrombus pictures before and after different treatments. Scale bar = 1 mm. Data are shown as mean ± SD (n = 3 biological replicates).

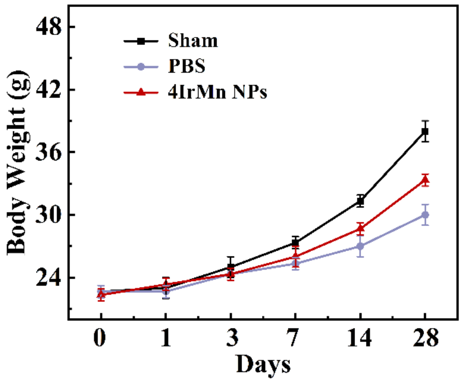

Figure S23. Body weights for different groups of mice at 0, 1, 3, 7, 14 and 28 days.

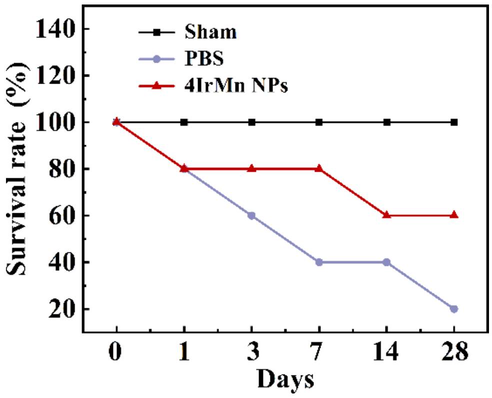

Figure S24. Survival rate for sham, PBS and 4IrMn NPs groups at 0, 1, 3, 7, 14 and 28 days.

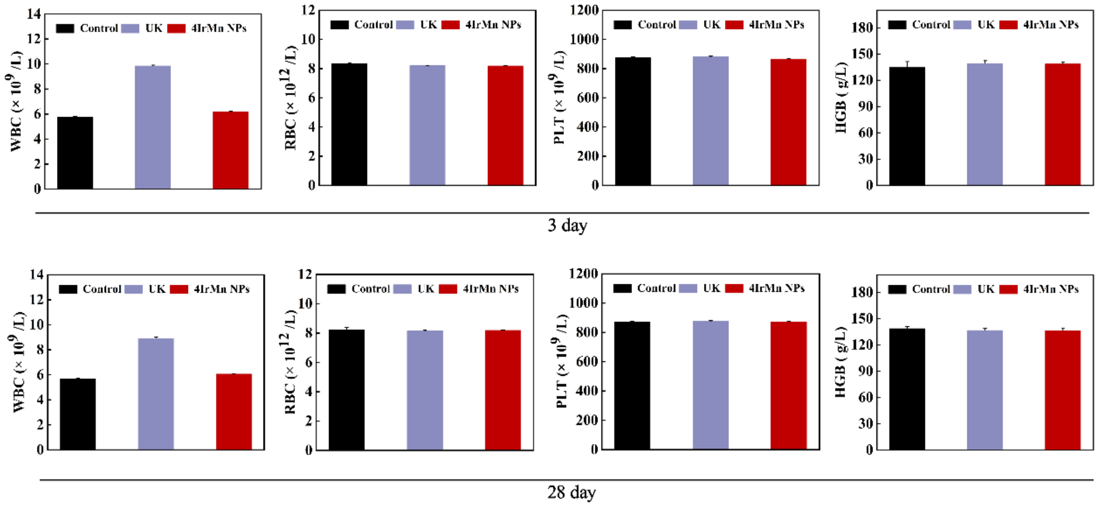

Figure S25. Blood test parameters for white blood cell (WBC), red blood cell (RBC), procalcitonin (PCT) and hemoglobin (HGB) blood cell count of the mice with different treatments at 3 days and 28 days.

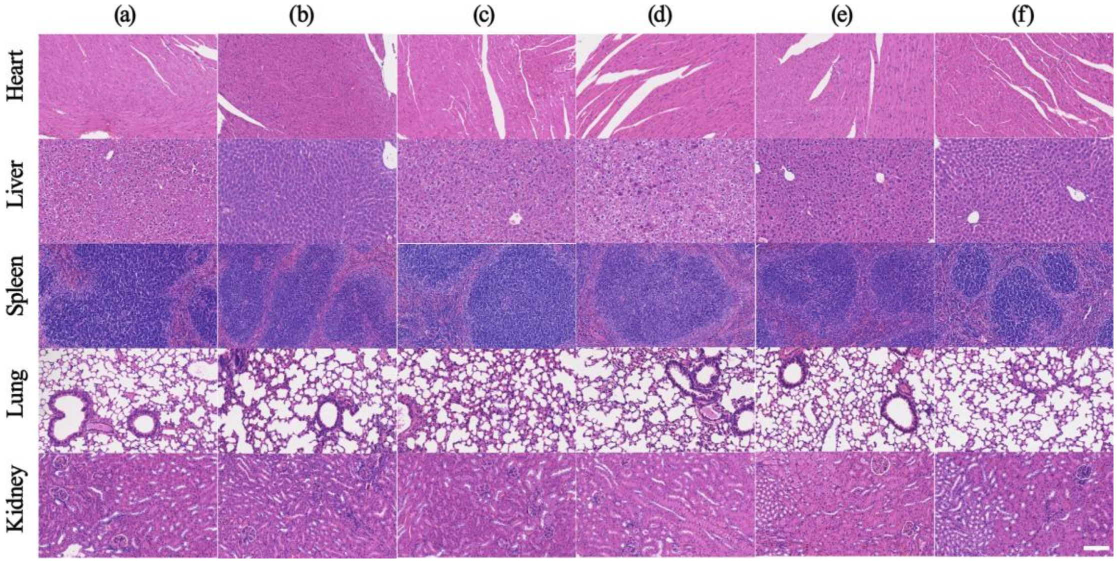

Figure S26. H&E staining of various organs from mice at the end of experiments a) with sham for 3 days, b) with MCAO for 3 days, c) with MCAO + 4IrMn NPs for 3 days, d) with sham for 28 days, e) with MCAO for 28 days, f) with MCAO + 4IrMn NPs for 28 days. Representative H&E-stained images from heart, liver, spleen, lung and kidney slices from untreated and 4IrMn NPs-treated mice. Scale bar, 100 µm.

Supplementary Table 1. Summary of ROS scavenging nanomaterials.

Materials	In vitro antioxidative activities	In vitro antioxidative activities
Melanin-based Nanoparticles[3]	79.4 ± 4.7% of $O_2^{\cdot -}$ at 25 μg mL^{-1}; 68.4 ± 2.5% of ·OH at 100 μg mL^{-1}; 85.2 ± 2.3% of ABTS at 100 μg mL^{-1};	25 mg kg^{-1} for acute kidney injury (AKI)
MSN-Ceria nanocomposites[4]	83% of H_2O_2 at 1.5 mM (210 μg mL^{-1}); 55% of $O_2^{\cdot -}$ at 0.12 mM (14 μg mL^{-1});	2.5-3.3 mg kg^{-1} for wound healing
Cu-TCPP nanosheets[5]	90% of $O_2^{\cdot -}$ at 5.5 μg mL^{-1};	0.8 mg kg^{-1} for AKI
Ceria nanoparticles[6]	90% of H_2O_2 at 0.6 mM (84 μg mL^{-1}); 40% of $O_2^{\cdot -}$ at 0.6 mM (84 μg mL^{-1}); 50% of ·OH at 0.6 mM (84 μg mL^{-1});	0.6 mg kg^{-1} for hepatic ischemia-reperfusion injury
Polydopamine[7]	~ 40% of $O_2^{\cdot -}$ at 0.6 mM (800 μg mL^{-1}); ~ 30% of H_2O_2 at 800 μg mL^{-1};	ischemic stroke
4IrMn NPs[this work]	74% of H_2O_2 at 300 μg mL^{-1}; 80% of DPPH at 300 μg mL^{-1}	ischemia-reperfusion injury

[1] X. Zheng, L. Wang, S. Liu, W. Zhang, F. Liu, and Z. Xie, Nanoparticles of Chlorin Dimer with Enhanced Absorbance for Photoacoustic Imaging and Phototherapy. *Adv. Funct. Mater.* **2018**, *28*, 1706507.

[2] Z. W. Wang, L. J. Li, W. J. Wang, R. L. Wang, G. Z. Li, H. Bian, D. X. Zhu, M. R. Bryce, Self-assembled nanoparticles based on cationic mono-/AIE tetra-nuclear Ir(iii) complexes: long wavelength absorption/near-infrared emission photosensitizers for photodynamic therapy. *Dalton Transactions* **2023**, *52*, 1595-1601.

[3] T. Sun, D. Jiang, Z. T. Rosenkrans, E. B. Ehlerding, D. Ni, C. Qi, C. J. Kutyreff, T. E. Barnhart, J. W. Engle, P. Huang, W. Cai, A melanin-based natural antioxidant defense nanosystem for theranostic application in acute kidney injury. *Adv. Funct.*

Mater. **2019**, *29*, 1904833.

[4] H. Wu, F. Li, S. Wang, J. Lu, J. Li, Y. Du, X. Sun, X. Chen, J. Gao, D. Ling, Ceria nanocrystals decorated mesoporous silica nanoparticle based ROS-scavenging tissue adhesive for highly efficient regenerative wound healing. *Biomaterials* **2018**, *151*, 66-77.

[5] L. Zhang, Y. Zhang, Z. Wang, F. Cao, Y. Sang, K. Dong, F. Pu, J. Ren, X. Qu, Constructing metal-organic framework nanodots as bio-inspired artificial superoxide dismutase for alleviating endotoxemia. *Mater. Horiz.* **2019**, *6*, 1682-1687.

[6] D. Ni, H. Wei, W. Chen, Q. Bao, Z. T. Rosenkrans, T. E. Barnhart, C. A. Ferreira, Y. Wang, H. Yao, T. Sun, D. Jiang, S. Li, T. Cao, Z. Liu, J. W. Engle, P. Hu, X. Lan, W. Cai, Ceria nanoparticles meet hepatic ischemia-reperfusion injury: the perfect imperfection. *Adv. Mater.* **2019**, *31*, 1902956.

[7] D. Wu, J. Zhou, Y. Zheng, Y. Zheng, Q. Zhang, Z. Zhou, X. Chen, Q. Chen, Y. Ruan, Y. Wang, Z. Chen, Pathogenesis-adaptive polydopamine nanosystem for sequential therapy of ischemic stroke. *Nat. Commun.* **2023**, *14*, 7147.
